# Averaged Propulsive Body Acceleration (APBA) Can Be Calculated from Biologging Tags That Incorporate Gyroscopes and Accelerometers to Estimate Swimming Speed, Hydrodynamic Drag and Energy Expenditure for Steller Sea Lions

**DOI:** 10.1371/journal.pone.0157326

**Published:** 2016-06-10

**Authors:** Colin Ware, Andrew W. Trites, David A. S. Rosen, Jean Potvin

**Affiliations:** 1 Center for Coastal and Ocean Mapping, University of New Hampshire, Durham, New Hampshire, United States of America; 2 Marine Mammal Research Unit, Institute for the Oceans and Fisheries, University of British Columbia, Vancouver, British Columbia, Canada; 3 Department of Physics, Saint Louis University, St. Louis, Missouri, United States of America; Coastal Carolina University, UNITED STATES

## Abstract

Forces due to propulsion should approximate forces due to hydrodynamic drag for animals horizontally swimming at a constant speed with negligible buoyancy forces. Propulsive forces should also correlate with energy expenditures associated with locomotion—an important cost of foraging. As such, biologging tags containing accelerometers are being used to generate proxies for animal energy expenditures despite being unable to distinguish rotational movements from linear movements. However, recent miniaturizations of gyroscopes offer the possibility of resolving this shortcoming and obtaining better estimates of body accelerations of swimming animals. We derived accelerations using gyroscope data for swimming Steller sea lions (*Eumetopias jubatus*), and determined how well the measured accelerations correlated with actual swimming speeds and with theoretical drag. We also compared dive averaged dynamic body acceleration estimates that incorporate gyroscope data, with the widely used Overall Dynamic Body Acceleration (ODBA) metric, which does not use gyroscope data. Four Steller sea lions equipped with biologging tags were trained to swim alongside a boat cruising at steady speeds in the range of 4 to 10 kph. At each speed, and for each dive, we computed a measure called Gyro-Informed Dynamic Acceleration (GIDA) using a method incorporating gyroscope data with accelerometer data. We derived a new metric—Averaged Propulsive Body Acceleration (APBA), which is the average gain in speed per flipper stroke divided by mean stroke cycle duration. Our results show that the gyro-based measure (APBA) is a better predictor of speed than ODBA. We also found that APBA can estimate average thrust production during a single stroke-glide cycle, and can be used to estimate energy expended during swimming. The gyroscope-derived methods we describe should be generally applicable in swimming animals where propulsive accelerations can be clearly identified in the signal—and they should also prove useful for dead-reckoning and improving estimates of energy expenditures from locomotion.

## Introduction

It is a common assumption in foraging theory that animals adopt strategies to maximize the ratio of energy gained over energy expended [1]—of which locomotion can be the major cost [2]. One easily measured proxy for energy expenditures associated with locomotion is acceleration [3,4], which can be collected using miniature data logging tags that incorporate three-axis accelerometers [5,6,7].

**Fig 1:**
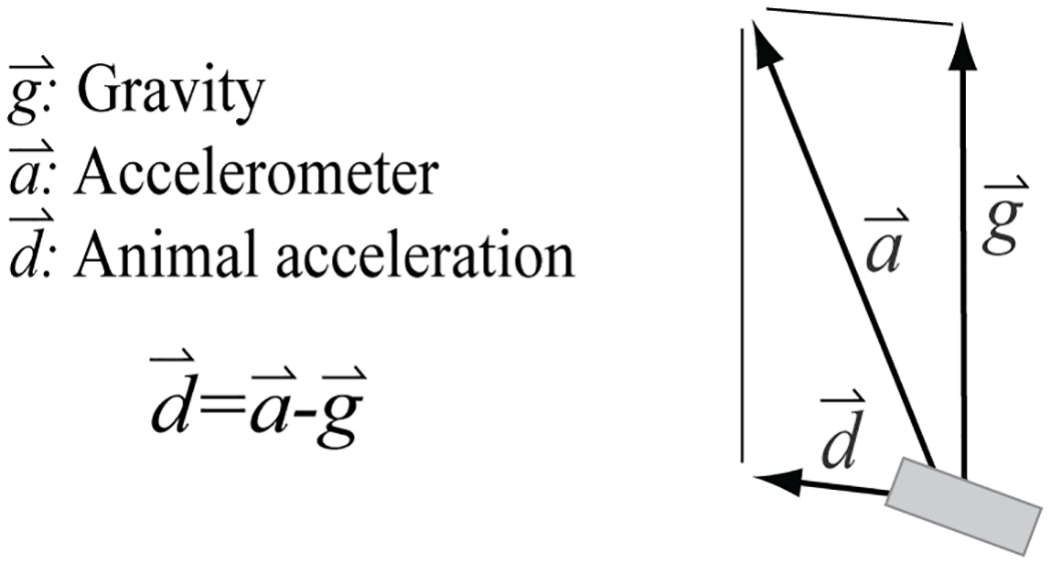
The measured accelerometer vector is the vector sum of gravity and dynamic tag acceleration. Acceleration due to gravity ($\overset{⃑}{g}$) is often called static acceleration, a constant at 9.8 m/s^2^ at the earth’s surface. Accelerations of an animal due to animal movements are dynamic ($\overset{⃑}{d}$). For large animals, accelerations of an animal’s mass due to locomotion tend to be substantially smaller than g, except for brief intervals. The measured acceleration ($\overset{⃑}{a}$) is the vector sum of $\overset{⃑}{d}$ and $\overset{⃑}{g}$.

Accelerometers measure the vector sum of acceleration due to gravity and animal movement (Fig 1). A widely used method for estimating animal accelerations generates a metric called Overall Dynamic Body Acceleration (ODBA; [3]). ODBA is calculated by subtracting a smoothed accelerometer signal from the raw signal (using a simple box filter to smooth the signals independently on each of three axes), then summing the absolute value of the results in each axis [3]. When there is little high frequency tag rotation, the smoothed signals will approximate the gravity signal (*static acceleration*) and the differences will approximate animal body acceleration (*dynamic acceleration*). ODBA is normally averaged over longer intervals (e.g., corresponding to a dive) to provide an estimate of activity, but it has also been used to provide sub-second estimates of energy expenditures [4]. A similar method to ODBA uses frequency filtering to separate the high frequency components of the accelerometer signals separately on each axis [8].

**Fig 2:**
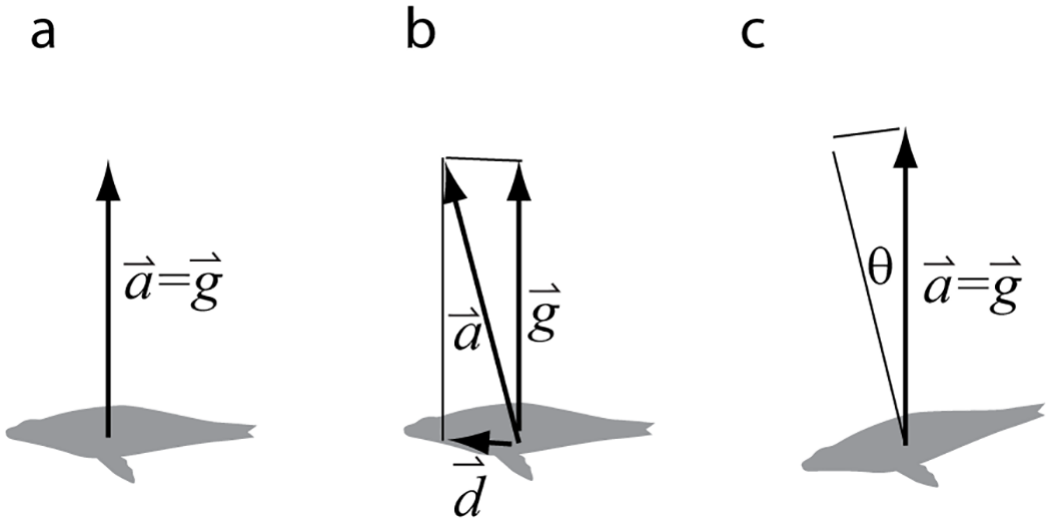
Schematics showing tag accelerometer measurements of animals that are a) resting, b) accelerating forward, and c) rotating. A resting animal (a) has measured acceleration $\overset{⃑}{a}$ equal to gravity $\overset{⃑}{g}$ (i.e., $\vec{a}\;=\;\vec{g}$). An animal that maintains a constant linear motion would give the same result (i.e., $\vec{a}\;=\;\vec{g}$). However, the acceleration signal changes (b) when the animal accelerates by $\;\overset{⃑}{d}$. This same change in the measured acceleration signal can occur if the animal rotates (c). For example, a linear acceleration of 1 m/s^2^ cannot be distinguished from a rotation of 5.82 deg. Hence, the behavior in (b) cannot be distinguished from the behavior in (c) unless there is an independent method for measuring orientation changes.

A key assumption of the ODBA method is that an animal is not rotating at a frequency above the cut-off frequency of the box filter, since a rotating gravity vector in the tag coordinate frame causes a change in the acceleration signals measured on the different axes in the absence of dynamic acceleration. Unfortunately, many animals swimming and diving in an aquatic environment do rotate, and it is not possible to separate linear forces from rotational forces using accelerometers alone (Fig 2). Hence, accelerometers will record changes in accelerometer signals caused by rotations, namely by changes in body pitch, rolls and yaw, that may be falsely interpreted as acceleration (Fig 2) [9].

**Fig 3:**
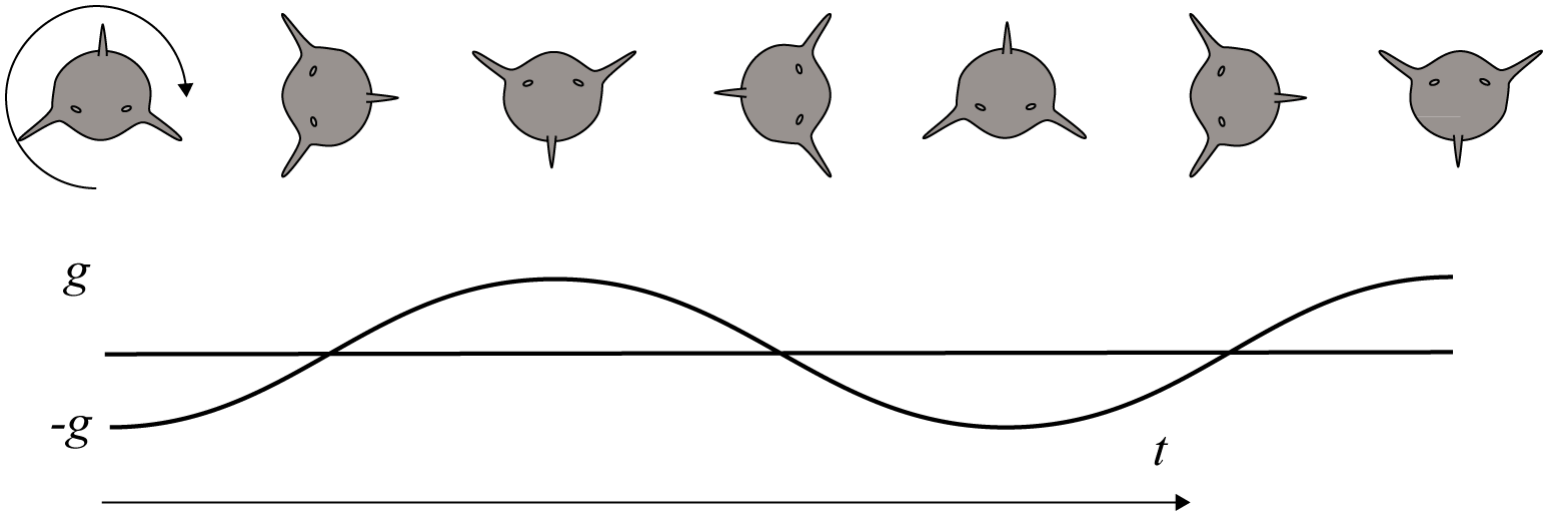
An example of a spinner dolphin rotating with a constant angular velocity about its own axis. The curve below shows the accelerometer signal from one axis of a three-axis accelerometer hypothetically placed at its center of rotation. With a 2 Π averaging window, the ODBA will amount to the average absolute value of the curve (6.24 m/s^2^) despite there being no dynamic acceleration.

In the hypothetical case of an animal such as a spinner dolphin (*Stenella longirostris*) that rotates about its own body axis at a constant angular velocity, a tag placed at its center of mass would measure a large ODBA (given a rotation rate greater than the box filter cut-off frequency) even though there is neither linear nor angular acceleration (Fig 3). ODBA thus includes the effects of animal rotation associated with extreme movements such as those of spinner dolphins. However, it also includes the effects of less extreme movements such as cetacean fluking, avian wing strokes, pinniped flipper strokes, and fish tail strokes.

Despite the shortcomings of ODBA, a number of empirical studies have shown that it provides a useful proxy for energy expenditure that correlates with rates of oxygen consumption for a number of species [10]. In addition, and as Gleiss et al. [4] point out, the primary acceleration proxies for propulsion are not necessarily forward acceleration in the direction of movement, as shown for the reef shark whose *lateral* accelerations (related to tail movements) were the largest contributor to ODBA.

A variation on ODBA uses a vector sum dynamic body acceleration rather than a simple addition of the axis components [11]. This has been called Vec DBA (VeDBA) and solves a problem with OBDA—namely that the summation of axis contributions overestimates accelerations occurring at oblique angles. However, Quasem et al. [11] found the correlations between VeDBA and rates of oxygen consumption were no higher, and were sometimes lower than for ODBA. Another variation—called partial DBA (PDBA)—uses a subset of accelerations to track heave and sway as a measure of activity, such as in hammerhead sharks [12].

A second common use of accelerometer signals is to estimate the orientation of an animal. Accelerometers can give a good indication of the direction of the center of the earth and can therefore be used to estimate an animal’s pitch and roll angle assuming that the body is rigid, the tag has a fixed position, and dynamic acceleration is small relative to *g*. Accelerometer data and magnetometer data (a digital compass) can be combined to estimate the 3D orientation of an animal together with its heading [5,13] A dead-reckoned trajectory can thereby be constructed if speed can be estimated, and the swimming animal is assumed to travel in the forward direction with respect to its long body axis [5,7,13,14].

The problem of decomposing changes in accelerometer signals due to gravity and animal movements can be resolved by adding gyroscopes to the instrument package [15]. Microelectricalmechanical systems (MEMS) gyroscopes measure the rate of rotation of an object about three axes and yield information that can be used to remove the confounding factor of rotations.

Our goal was to apply a method for estimating forward (in the direction of swimming) body accelerations taking advantage of gyroscope data combined with accelerometer data to calculate linear dynamic acceleration. We hoped to use forward accelerations as indicators of the power and frequency of swimming strokes for Steller sea lions (*Eumetopias jubatus*) and to determine how well derived measures correlated with swimming speed. A second goal was to determine whether forward linear body accelerations could also be used to estimate energy expenditures after accounting for fluid dynamic drag―a goal enabled by a new metric, the Averaged Propulsive Body Acceleration (APBA). A good correlation between measured accelerations and estimated drag would provide confidence that the measurements of acceleration are related to the energetic cost of locomotion. Finally, we compared APBA with ODBA in terms of how well each correlated with swimming speed (which can be taken as a proxy for energy expended)—although we recognize that ODBA was not designed to predict speed.

Sea lions provide an excellent model to test the ability of methods to estimate dynamic body accelerations arising from propulsive forces. Steller sea lion swimming propulsion comes almost entirely from intermittent brief strokes of the fore flippers, and their swimming mechanism can be characterized as a rigid body with wings, unlike true seals whose propulsion comes from the hind flippers combined with whole body undulations [16]. The animal glides between strokes with the fore flippers held close to the body [17,18,19]. At the start of the stroke cycle, the animal brings the fore flipper forward and raises it. The main propulsive force comes from a downward power stroke followed by rotation of the flipper to become more at right angles to the rostral-caudal axis, and a final paddle stroke to the gliding position. The power stroke typically only lasts a fraction of a second and is followed by a glide of 1–3 seconds [18]. In the study reported here, we used trained Steller sea lions who could perform directed controlled tasks to evaluate methods for estimating propulsive acceleration and related metrics.

## Materials and Methods

We attached tags to four trained adult female Steller sea lions (F00YA, F97HA, F00BO and F97SI) housed at the University of British Columbia’s Open Water Research Station (Burrard Inlet on the coast of British Columbia, Canada. Coordinates: 49.292, -122.891) to determine the relationship of our proposed metric to a known behavior. Trials were run on one day in July, three days in November and one day in December 2013. All research was conducted under UBC Animal Care Permit #A11-0397.

The sea lions were weighed and measured for body length (Table 1). Their surface areas were estimated by fitting a power curve to published data [20] for similarly-sized animals and extrapolated or interpolated based on their measured body mass. The animals were trained to swim alongside a 6.4 m research vessel with an outboard jet engine, at the speed of the vessel, in exchange for food rewards. The trials were run in two directions east to west and west to east to compensate for local currents (estimated < 0.5 kt).

**Table 1:** Animal ID, age, mass, length and surface area of 4 adult female Steller sea lions that participated in the study. Surface areas estimated by fitting a power curve to published data [20] for similarly-sized animals and extrapolated or interpolated based on their measured body mass.

Animal ID	Age (y)	Mass (kg)	Length (m)	Maximal body diameter (m)	Surface area (m^2^)
F00YA	13	209.5	228.7	0.51	3.27
F97HA	16	171.5	152.0	0.49	2.92
F97SI	16	220.5	223.7	0.52	3.37
F00BO	13	142.5	205.0	0.45	2.63

The boat was run at 1.11 m/s (4km/h), 1.67 m/s (6km/h), 2.22 m/s (8km/h) and 2.78 m/s (10km/h) with the goal of obtaining 45+ seconds of constant speed subsurface horizontal straight-line swimming at each speed. These speeds were not exact and actual boat speed (± current) was obtained using a Garmin eTrex 20 GPS data logger recording every 5s. The sea lions tended to keep their eye on the trainers while swimming, and to propel themselves higher than necessary for simple directional travel. They also often fell back after receiving fish rewards at higher speeds. To correct for this, the fall back distance was estimated for each dive speed and to correct the speed.

### Biologging tag

We used two Loggerhead Instruments OpenTags each containing an Analog Devices ADX345 3-axis digital accelerometer, a Honeywell 3-axis magnetometer, HMC5883L, an InvenSense IMU-3000 3-axis gyroscope, and a Measurement Specialties MS5803-30BA miniature 30 bar pressure sensor, as well as temperature recorder that we did not use. The tags were set to record all values at 50 Hz.

**Fig 4:**
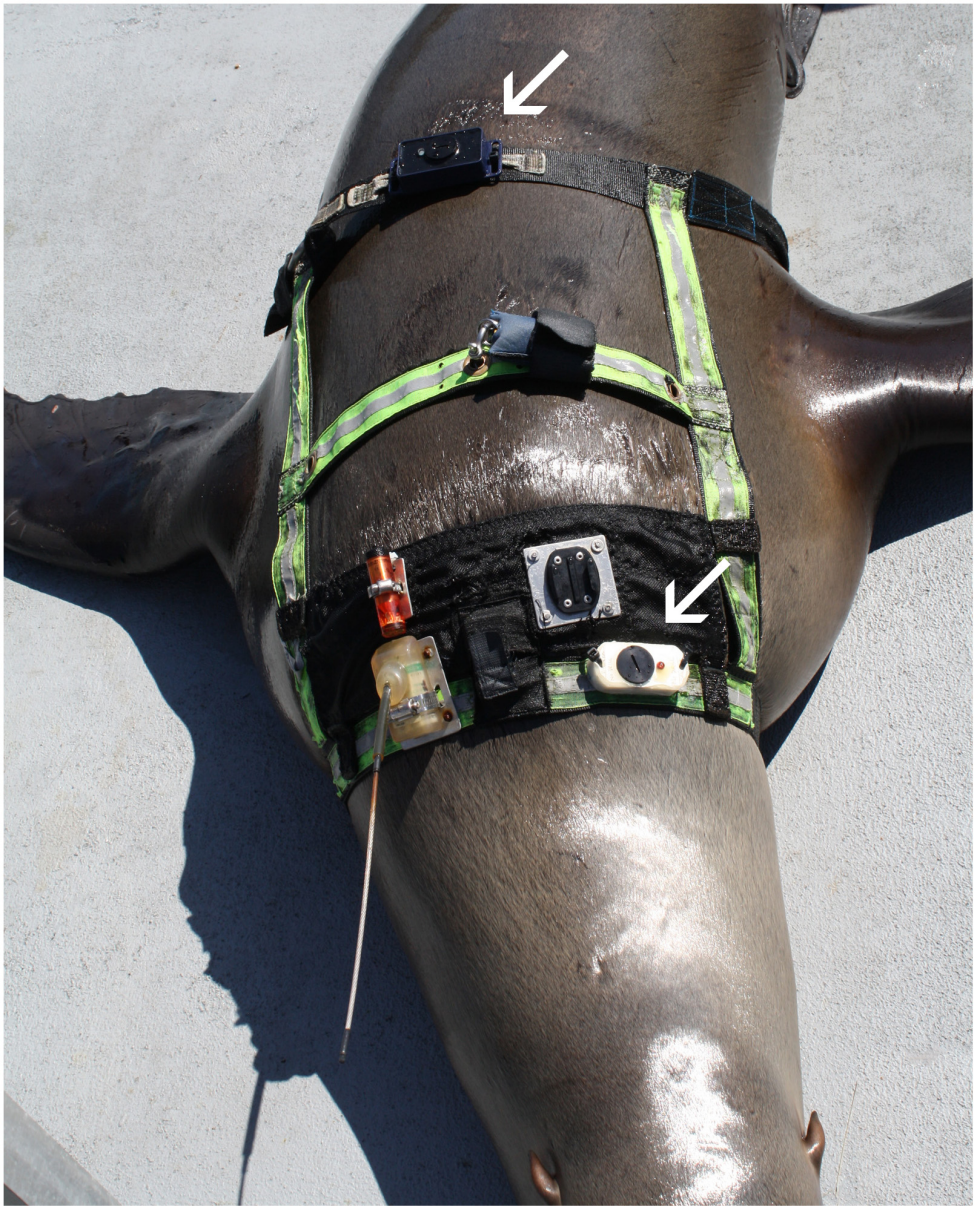
Harness attachment on an adult female Steller sea lion. The two OpenTags are shown by the arrows. Only data from the more rostral were used for the study.

Two OpenTags, a time-depth recorder, and a VHF-tracking device were affixed to each animal via a specially-designed, tight-fitting harness (Fig 4). This harness is worn by the animals on a daily basis when the animals swim in open water. Two tags were attached, but only the more rostral tag provided useful data because the strap caudal to the flippers became loose during swimming and the data revealed large resultant oscillations.

### Gyro-Informed Dynamic Acceleration (GIDA)

Our method differed somewhat from that of prior researchers [15,21,22]. We used a complementary filter similar to that of [23] and [24] instead of a Kalman filter [15,21] or a gradient descent algorithm [22] to correct drift in the estimated gravity vector. For brevity, we refer to the gyroscope method as GIDA ― Gyro-Informed Dynamic Acceleration.

GIDA is calculated as follows: If the gravity vector $\overset{⃑}{g}$ can be estimated in tag coordinates then dynamic accelerations can be obtained simply by subtracting $\overset{⃑}{g}$ from the measured acceleration vector $\vec{a}\;(\vec{d}\;=\;\vec{a}\;–\;\vec{g})$. Producing a continuous estimate of $\overset{⃑}{g}$ starts with collecting data on a stationary tag in order to provide an initial direction of $\overset{⃑}{g}$ in tag coordinates $\;(\vec{d}\;=\;0$, $\vec{g}\;=\;\vec{a}$). Iterating forward, an estimated gravity vector g⃑' is counter-rotated on every (20 ms) time step using the 3-axis gyro signal. In principle, this should produce a vector that tracks actual gravity assuming perfectly accurate instruments. However, inaccuracies in the gyro signal will cause this vector to drift from the true gravity vector. A complementary filter is used to correct for this drift [23]. Dynamic acceleration is computed for the entire track using $\left(\vec{d}\;=\;\vec{a}\;–\;\vec{g}\prime \right)$. Further details of this method are given in Appendix 1.

**Fig 5:**
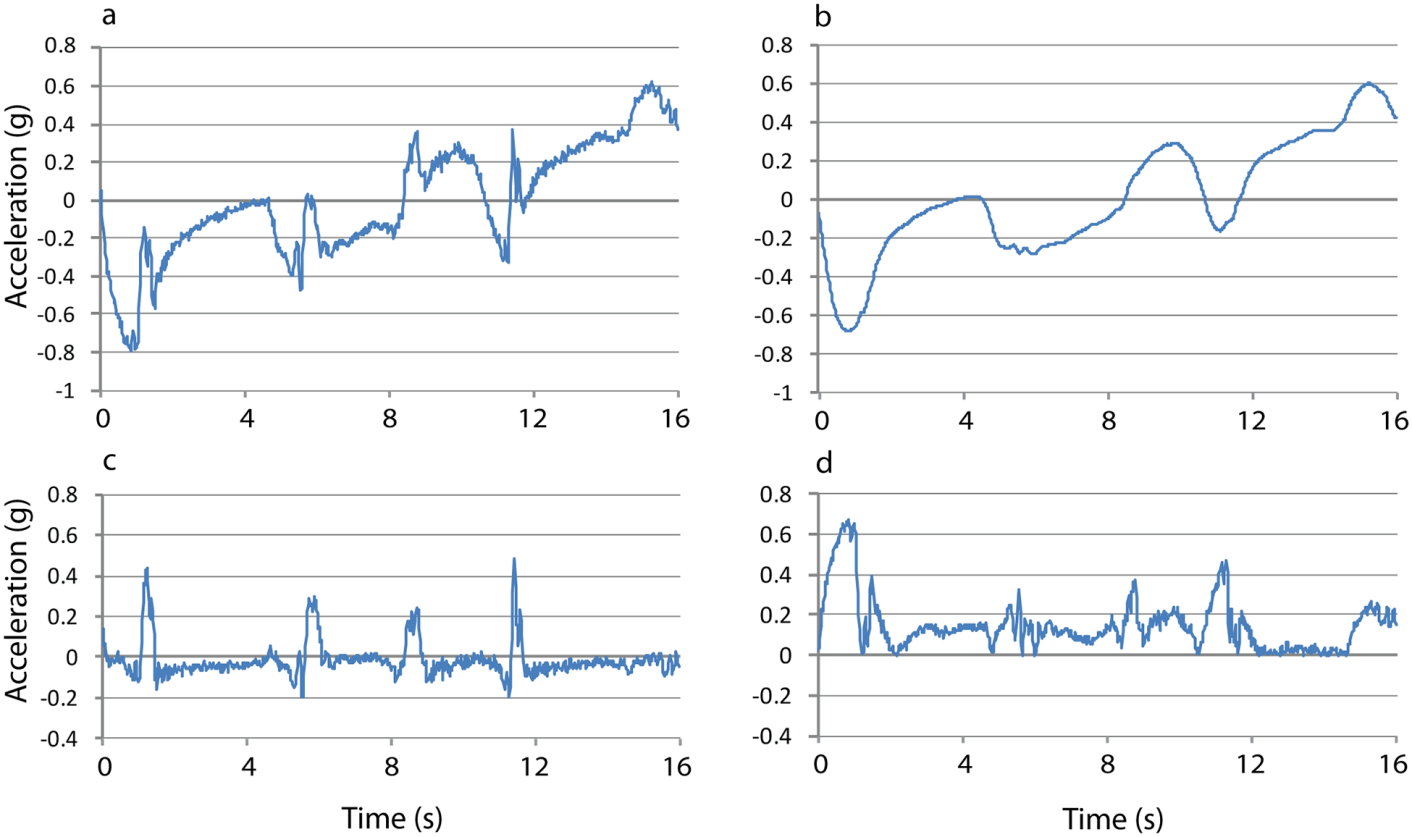
The raw x-axis acceleration signal (a) and estimated (gyro-informed) acceleration due to gravity (b) of a female Steller sea lion that stroked four times during a single dive. The difference between the curves in panels a and b equals the forward acceleration in the tag’s x-axis (GIDA) (c). The result from subtracting the smoothed accelerometer signal from the unfiltered for the tag’s x-axis (d). (Animal F00BO, July 30^th^, 6 kph).

**Fig 6:**
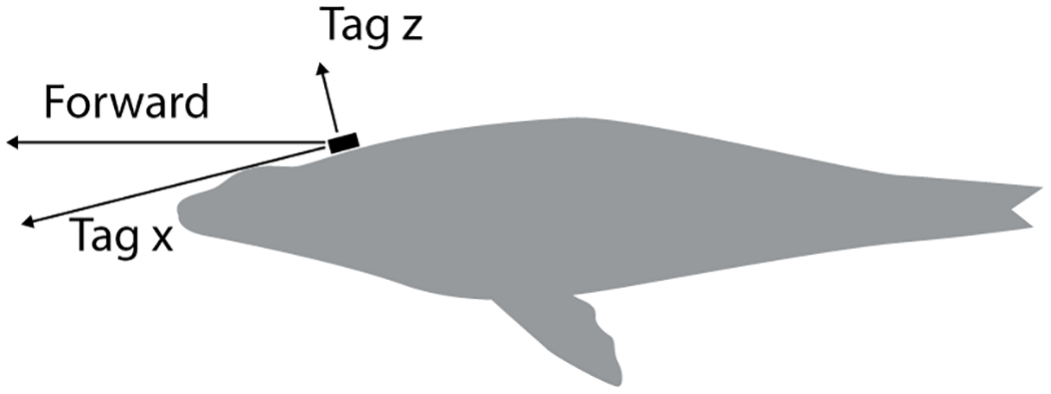
The tag axes are shown relative to the animal.

Fig 5 illustrates the result of applying this method to data from a single short dive. For clarity, we show only the x-axis signal (this was the axis that roughly pointed in a forward direction; Fig 6). Fig 5a shows the raw x-axis acceleration signal, and Fig 5b the corresponding x-component of the estimated gravity vector g⃑' in which the animal is pitched down at the first part of the dive and pitched up at the end of it. Fig 5c shows the result of subtracting g⃑' from the accelerometer signal (thus yielding GIDA), thereby revealing the net acceleration generated during fore-flipper stroking, followed by the decelerations characteristic of the glide phase in which the flippers are abutted against the body (and thereby producing no thrust). Subtracting a smoothed accelerometer signal from the unfiltered signal (the first step of the ODBA method) also shows the four individual flipper strokes, but the signal is less clear (Fig 5d).

### ODBA calculation

In the ODBA method, static acceleration is estimated by using a box filter on each axis of a three-axis accelerometer [3]. According to an analysis by Shepard et al. [6], the optimal running mean interval for ODBA should be longer than the stroke period, and should be a minimum of 3 sec. The animals in our study had a stroke period that varied between ~ 2 sec at high speed and 3 sec at low swimming speeds. We therefore used a running mean filter width of 3.0 sec.

### APBA-based Calculations

**Fig 7:**
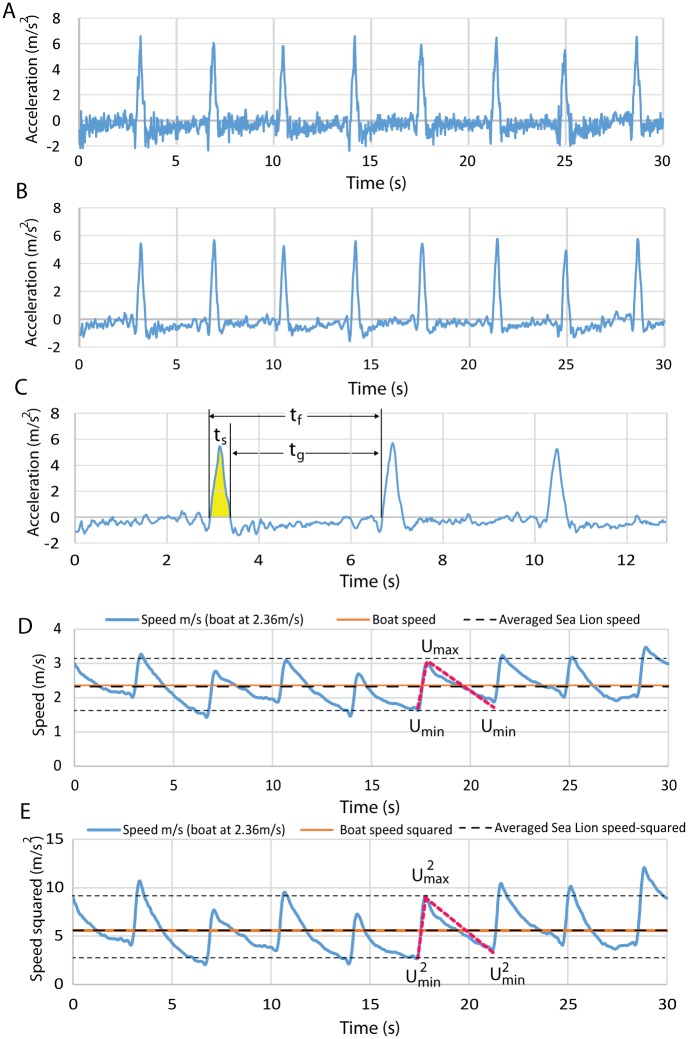
A series of strokes from an animal swimming at approximately 2.36 m/s. A) Forward acceleration (GIDA) sampled at 50 Hz. B) GIDA smoothed to 10 Hz. C) The area under the curve in the interval *t*_*s*_ is the gain in speed from a stroke. The glide duration is *t*_*g*_ and the duration of an entire stroke-glide cycle is *t*_*f*_. D) Speed obtained by integrating the smoothed acceleration data. The model is indicated by the red dashed line. E) Speed squared from the same data. Horizontal dashed lines represent the averaged MIN and MAX speeds.

We estimated the tag was tilted forward by approximately 20 degrees (Fig 6) which means that acceleration in a forward direction using the x and z tag coordinates was: a_f_ = cos(20)a_x_ + sin(20) a_z._. Our metric of Averaged Propulsive Body Acceleration (APBA) is defined as the gain in speed, U_max_−U_min_ (Fig 7d) in the direction of travel from a swimming stroke divided by the peak-to-peak inter-stroke interval (*t*_*f*_). This provides a measure commensurate with ODBA (its unit is acceleration) which can be used in estimates of metabolic expenditure and also, we believe, dead reckoning. APBA can be used in the computation of both time-averaged drag and propulsion force (thrust) as illustrated here and derived in Appendix 2.

In contrast to GIDA which yields near-instant acceleration at the temporal resolution of the recording device, APBA is connected to the time-averaged accelerations <a>_ts_ and <a>_tg_ sustained during the stroke and glide phases (assuming no net acceleration) as follows:

$$(APBA)=\frac{t_{s}}{t_{f}}{\langle a\rangle }_{ts}=\frac{t_{g}}{t_{f}}(-1){\langle a\rangle }_{tg}$$ (1)

The time intervals *t*_*s*_, *t*_*g*_ and *t*_*f*_ correspond to the duration of the foreflipper stroke phase, glide phase, and one complete stroke-glide cycle respectively (see also Fig 7c). In this, and the following equations, the use of a triangular bracket indicates a time averaged variable (see Eq A2-7 in Appendix 2).

To identify peaks in forward acceleration corresponding to flipper strokes, the data were first smoothed to 10 Hz using a box filter. A second process identified local maxima > 0.06 g for the slowest speed (4 kph) and > 0.15 g for higher speeds within a 400 ms moving window.

**Fig 8:**
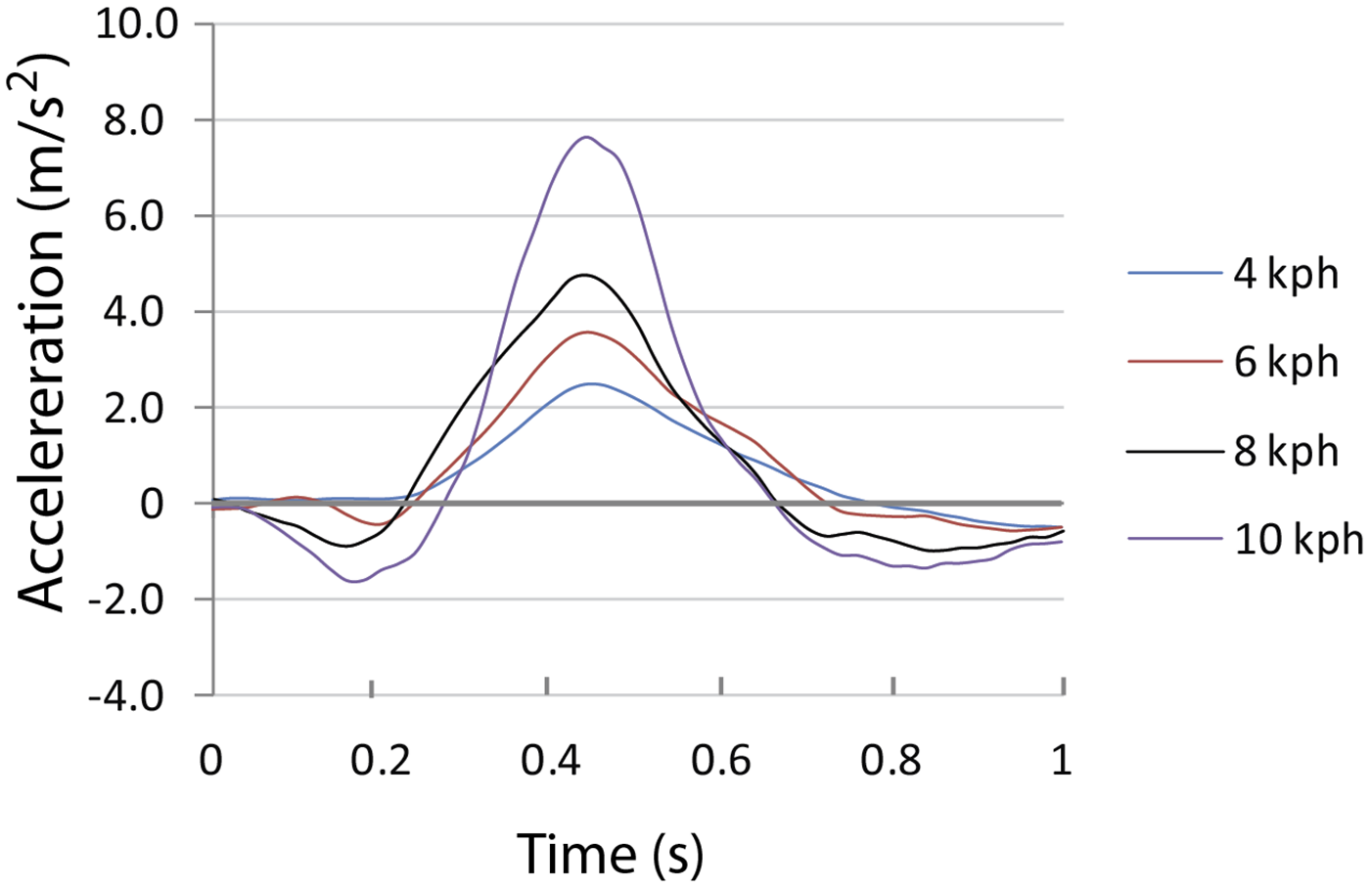
Estimated acceleration in direction of travel at different speeds for one Steller sea lion (F97SI). Averaged from 10 strokes each.

In order to estimate the gain in speed from flipper strokes, the area under the acceleration curve was integrated in a 400 ms window centered on the peak. The window width was chosen to be the typical duration of forward acceleration. Fig 8 shows the averaged profile (based on ten strokes) at each of the four speeds attained by one animal (F97SI). These were normalized to be coincident at the peak accelerations and show that the strokes were brief pulses of propulsive force, with a half width of the acceleration profile of around 300 msec. They also show that the strokes become narrower and produced larger accelerations at higher speeds.

During the stroke phase, the time-averaged thrust from a foreflipper stroke is given by the averaged acceleration multiplied by the combined mass of the animal and entrained water, plus the time-averaged drag:

$${\langle T\rangle }_{ts}=\left(M_{animal}+M_{entrained}\right){\langle a\rangle }_{ts}+{\langle D\rangle }_{ts}$$ (2)

Both *T* and *D* are defined as positive but <a> is allowed to be either positive or negative. Here *D* is understood as body drag, i.e., without flipper contributions as the latter act as propulsors. Thus, the thrust *T* in Eq 2 is “effective” thrust, i.e., as would be obtained via subtraction of flipper drag from “true” thrust if both happen to be known (a difficult task, as further discussed in [25]. Effective thrust is sufficient for the complete calculation of the trajectory (Eq 2), but true thrust is required for estimating the metabolic expenditures. In the absence of the latter, and per standard practice in aircraft design [26,27,28], we used a “propeller” efficiency factor used in metabolic power estimates to account for flipper drag losses (both longitudinally and laterally) as further discussed below (Eq 9). Parameter *M*_*entrained*_ corresponds to the so-called *entrained added mass*, used to factor-in the effects of water slugs co-accelerated longitudinally along with the animal. Here *M*_*entrained*_ *~ 0*.*05 M*_*animal*_ per other studies [20,29]. This added mass term is the first of two such terms used in this analysis.

The time-averaged equation of motion during the glide phase look similar, but without a flipper thrust term, given the absence of foreflipper stroking:

$$\left(M_{animal}+M_{entrained}\right){\langle a\rangle }_{tg}=-{\langle D\rangle }_{tg}$$ (3)

Here *D* is again understood as body drag, a reasonable assumption if the flippers are assumed to be firmly tucked against the body (as they appear *in vivo*) thereby contributing little extra drag.

On the other hand, time-averaging the equation of motion over an entire stroke-glide cycle (*t*_*f*_) yields

$$\left(M_{animal}+M_{entrained}\right){\langle a\rangle }_{tf}={\langle T\rangle }_{tf}-{\langle D\rangle }_{tf}=0$$ (4)

The last step on the right-hand-side results from the zero time-averaged acceleration characterizing an animal following a boat cruising at constant speed. Although the tag data (Fig 7) suggest that this will not be the case for *all* stroke-glide cycles, the constraint will hold to a good approximation for at least many such cycles, as suggested by Fig 7d.

Connecting the value of APBA to drag and thrust goes as follows. For the glide phase, merging Eqs 1 and 3 yields a measurement of the drag produced (see details in Appendix 2):

$${\langle D\rangle }_{tg}=\frac{t_{f}}{t_{g}}\left(\text{APBA}\right)\left(M_{animal}+M_{entrained}\right)$$ (5)

To carry out the calculation of flipper thrust via Eq 4, we assume that stroke and glide body drag are nearly the same over each stroke and glide cycle―namely *<D>*_*ts*_ *~ <D>*_*tg*_― given the similarity of the time-averaged swimming speed as further explained below.

From this assumption, and along with merging Eqs 1 and 2 and the definition of APBA, one gets the following computation of thrust, as averaged over a full stroke-glide cycle (Appendix 2):

〈T〉tf=(APBA)(Manimal+Mentrained)(1−tstf) (6)

Aiming for a comparison with otariid body drag discussed in past studies [19,20,30], the drag force is further expanded as follows during each stroke and glide phase:

$$\begin{array}{l} \langle D\rangle =\gamma \frac{1}{2}\rho S_{wetted}C_{D}{}^{body}\langle U^{2}\rangle +k\rho (TAD)\langle U\rangle \omega \\ \sim \gamma \frac{1}{2}\rho S_{wetted}C_{D}{}^{body}U_{boat}{}^{2}+k\rho (TAD)U_{boat}\omega \end{array}$$ (7)

The first term in the right-hand-side, is the so-called *body form drag*, expressed in terms of the density of seawater (*ρ* = 1027kg/m^3^), body wetted area (*S*_*wetted*_) (Table 1) and the steady-state drag coefficient *C*_*D*_^*body*^ (0.0054; Feldkamp 1987b) [19]. The factor γ (1 ≤ γ ≤ 5) accounts for wave drag effects that arise when the animals swim near the surface [27,28,31,32]. Eq 7 also allows for the body drag to be different in the stroke and glide phases if *<U>* and *<U*^*2*^*>* are different in each phase. But in the context of the experiment described here, both speed averages turn out the same and nearly equal to the boat’s *U*_*boat*_ and *U*_*boat*_^*2*^ (as further argued below). From this follows the second line in Eq 7 and the fact that body drag changes little in both phases.

The second term in Eq 7 represents the ability of the harness at generating drag because of the flutter of the rear strap (Fig 4), which propels extra slugs of water both vertically and horizontally. This is a second source of added mass, i.e., in addition to the entrained mass term (*M*_*entrained*_) codified in Eqs 2 and 3, but one that would affect only harnessed swimmers outfitted with loose straps carrying bulky hardware. This term is derived by estimating the rate of momentum gained by the water surrounding the strap, namely *U dM*_*water*_*/dt* ~ *kρTADUω*, a product of the angular flutter frequency of the strap (*ω*; radians/s) (measured by the accelerometer affixed to the aft strap), seawater density, strap length (*D*), width (*T*) and strap oscillation amplitude (*A*). The value of coefficient *k* expresses the percentage of the mass disturbed by the strap, as parameterized in units of *ρTAD*. With all other inputs known (*U*_*boat*_ ~ 1–3m/s, D = 1.5m, T = 0.1m, A = 0.04m and ω at 44.5 radians/s, and γ = 1.0 (swimming at depth)) parameter *k* is determined from the tag data via Eq 5, which incidentally accounts for both sources of added mass, i.e., via *k* and *M*_*entrained*_. Note that with the latter already fixed at *M*_*entrained*_ */M*_*animal*_ *~ 0*.*05* [20,29], using Eq 5 to get a value of *k* accounts for all other sources of added mass co-moving with the animal.

Approximating a sea lion’s time-averaged speed and speed-squared with the boat’s *U*_*boat*_ and *U*_*boat*_^*2*^ as done in Eq 7 needs to be justified and quantified, along with the claim that a sea lion’s drag is quantitatively the same in both gliding and stroking phases. Clearly, time-averaging (over each stroke-glide cycle) the speed of an otariid trained to strictly follow a boat moving at constant velocity shall always yield a value equal to that of the boat’s, regardless of the specifics of the measured speed profile. This is slightly different in the experimental trials shown in Fig 7d, where the matches occur only over a subset of the cycles, and with small deviations over the rest, i.e., when the animal sometimes gets somewhat ahead of the boat (see cycle 20-25s) or behind it (as in cycle 5-10s). Thus, approximating *<U>* with *U*_*boat*_, as done in the added mass term in Eq 7 is accurate, but only as averaged over several stroke-glide cycles.

On the other hand, calculating the average *<U*^*2*^*>* is more problematic since it also depends on the shape and other details of a speed profile, such as the values of *U*_*max*_ and *U*_*min*_ marking the end of the stroke and glide cycles respectively. It is such dependence to profile details that lead to what is commonly known: namely, that the average of the square of a function is not necessarily equal to the square of its average. Another complication is that the profile is not the same on a (full) cycle-to-cycle basis. Therefore, assessing the errors connected with assigning *<U*^*2*^*>* to *U*_*boat*_^*2*^ is done by modeling the speed profiles themselves with the linear time functions represented by the dashed red line in Fig 7d (see also Eqs A2-1–A2-4). Clearly, such a profile does not match *all* glide-stroke cycles, but does adequately represent most cycles nevertheless. Among the useful properties that this type of linear profile offers is the time-averaged of the otarid’s speed and speed-squared (*U* and *U*^*2*^), which can be calculated as given exactly by *<U>*_*ts*_ = *<U>*_*tg*_ = *U*_*boat*_ and *<U*^*2*^*>*_*ts*_ = *<U*^*2*^*>*_*tg*_ = *U*_*boat*_^*2*^ *+ (U*_*max*_*−U*_*min*_*)*^*2*^*/12* (see Appendix 2 for details). And so, it would appear that it is indeed reasonable to assume the drag as being similar in both gliding and stroking phases if the speed averages are the same in each phase. Moreover, this result suggests that approximating *<U*^*2*^*>* with *U*_*boat*_^*2*^ incurs small errors of only 5% or less for the typical speed profiles shown in Fig 7d.

Finally, the metabolic expenditure *<P>* of an animal is given by the sum of the resting and swimming metabolic expenditures. Resting metabolic expenditure (Watts) is a power function of the mass of the animal [2,33] estimated herein via

$${\langle P_{rest}\rangle }_{tf}=2\cdot 4{\left(M_{animal}\right)}^{0.75}$$ (8)

The metabolic expenditure from swimming is given by the product of time-averaged thrust and the swimming speed divided by the metabolic efficiency (*μ*_*m*_) and the propulsive efficiency (*μ*_*p*_; also called propeller efficiency) [27,28].

$${\langle P_{swim}\rangle }_{tf}=\frac{{\langle T\rangle }_{tf}U_{boat}}{\mu_{m}\mu_{p}}$$ (9)

Here again the swimming speed has been approximated by the boat’s speed. Parameter *μ*_*m*_ characterizes the chemical energy converted into mechanical energy when using muscles; and *μ*_*p*_ accounts for the extra mechanical energy needed, during a propulsive stroke, by the flippers for moving fluid vertically in addition to horizontally (in other words, this is where flipper drag must be taken into account explicitly). Feldcamp ([19] Fig 8) found that for California sea lions, propulsive efficiency is a function of speed, being substantially greater at higher speeds than at low speeds. We derived a *μ*_*p*_ function by fitting a quadratic curve to his published data.

## Results

**Fig 9:**
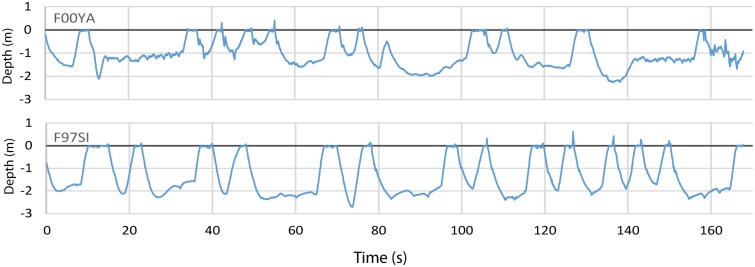
Samples of dive profiles for two animals.

We selected dives (periods of subsurface swimming) for analysis that met the following criteria: a duration of 12 s or more, no wanderings away from the task noted by the trainer, a roughly constant swimming depth, and a GPS speed approximating the target speed. We obtained data from a total of 133 dives (between 25 and 48 dives per sea lion). We obtained more dives at the slower speeds because the animals frequently gave up at the higher speeds. It took several attempts to obtain speeds approximating 10 kph for three of the animals, and one animal never met the test criteria at that speed. The pressure sensor data showed the animals swimming at depths approximating 1.5–2.0m, i.e., close to the 3x body diameters at which form drag is minimal and where γ = 1 ([32]; Fig 9).

**Fig 10:**
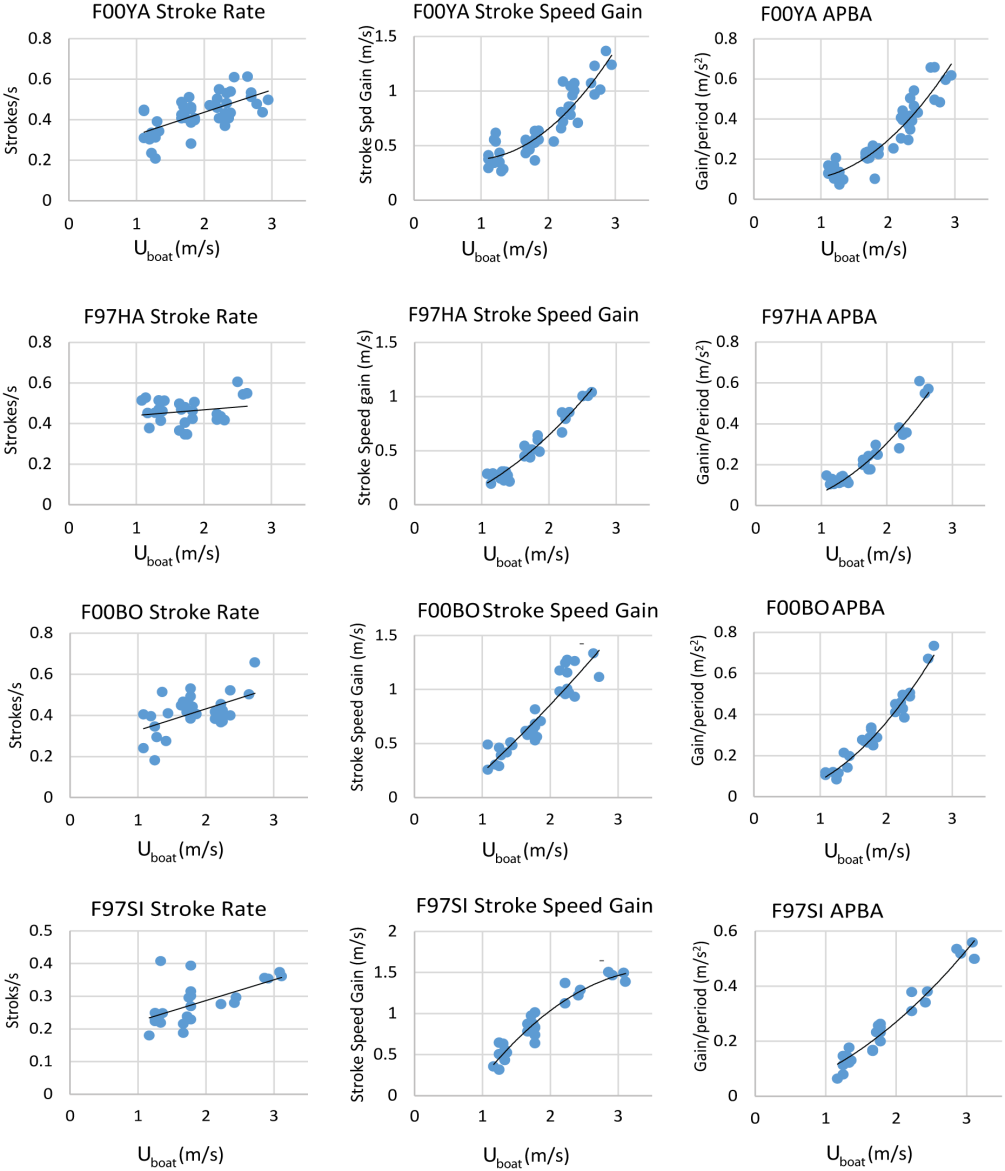
The left hand column illustrates the relationship between stroke rate and swimming speed for the four animals. Each point represents data from a single dive. The middle column illustrates the relationship between the average speed gain during single strokes and boat speed for the four animals. The right hand column illustrates the relationship between the stroke speed gain multiplied by the stroke rate (APBA).

We processed all of the data from all of the selected dives to identify peak forward accelerations during strokes and computed the mean gain in speed for the strokes in each dive meeting our criteria. We also computed the mean stroke rate for all dives. Fig 10 (column 1) shows how the stroke rate varied with speed for the four animals. All regression equations and r^2^ values for the figures are provided in Table 2. For three of the four animals, there was a clear, although weak positive correlation between stroke rate and speed (F00YA, r^2^ = 0.46; F00BO, r^2^ = 0.28; F97SI, r^2^ = 0.36). For one animal (F97HA, r^2^ = 0.04), the stroke rate only increased at the highest speeds. Much higher correlations were obtained for the relationship between average speed gain per stroke and speed as illustrated in the second column of Fig 10 (F00YA, r^2^ = 0.84; F97HA, r^2^ = 0.95; F00BO, r^2^ = 0.85; F97SI, r^2^ = 0.92).

**Table 2:** Regression equations and r^2^ values for all scatter plots.

	F00YA	F79HA	F00BO	F97SI
Fig 10				
Stroke Rate	y = 111x+0.2155r^2^ = 0.46	y = 0.0283x +0.411r^2^ = 0.043	y = 0.104x+0.223r^2^ = 0.28	y = 0.0635x+0.160r^2^ = 0.36
Stroke Spd Gain	y = 0.226x^2^+0.401x+0.558, r^2^ = 0.84	y = 0.119x^2^+0.116x+0.06, r^2^ = 0.95	y = 0.041x^2^+0.507x+0.318, r^2^ = 0.85	y = 0.194x^2^+01.40 x+0.985, r^2^ = 0.92
APBA	y = 0.073x^2^+0.006xr^2^ = 0.88	y = 0.089x^2^+0.026xr^2^ = 0.92	y = 0.10x^2^+0.02xr^2^ = 0.96	y = 0.042x^2^+0.051xr^2^ = 0.95
Fig 11	y = -0.115x^2^+1.617xr^2^ = 0.57	y = -0.727x^2^+3.625 xr^2^ = 0.04	y = 0.586x^2^+144xr^2^ = 0.90	y = 0.16x^2^+64xr^2^ = 0.833
Fig 12	y = -20.68xr^2^ = 0.87	y = -17.21xr^2^ = 0.88	y = -16.67xr^2^ = 0.93	y = -16.7xr^2^ = 0.92
Fig 13	y = -2.28xr^2^ = 0.87	y = -20.68xr^2^ = 0.88	y = -20.68xr^2^ = 0.87	y = -20.68xr^2^ = 0.87
Fig 14	y = 89.65x+98.4r^2^ = 0.86	y = 86.2x+11.2r^2^ = 0.96	y = 83.9+42.1r^2^ = 0.90	y = -90.9x+229.6r^2^ = 0.87
Fig 15	y = -1.34x^3^+12.32x^2^-26.47x +21.27, r^2^ = 0.89	y = 2.01x^3^-5.57x^2^+4.60x +4.00, r^2^ = 0.91	y = 5.98x^3^-29.05x^2^+50.14x -23.75, r^2^ = 0.95	y = 1.89 x^3^-9.19x^2^17.74x -7.27, r^2^ = 0.97

Three of the four animals had a stronger correlation between swim speeds and the APBA metric (mean speed gain divided by mean inter stroke interval) (F00YA, r^2^ = 0.88; F00BO, r^2^ = 0.96; F97SI, r^2^ = 0.95) and the correlation was the same for one of the sea lions (F97HA, r^2^ = 0.92).

**Fig 11:**
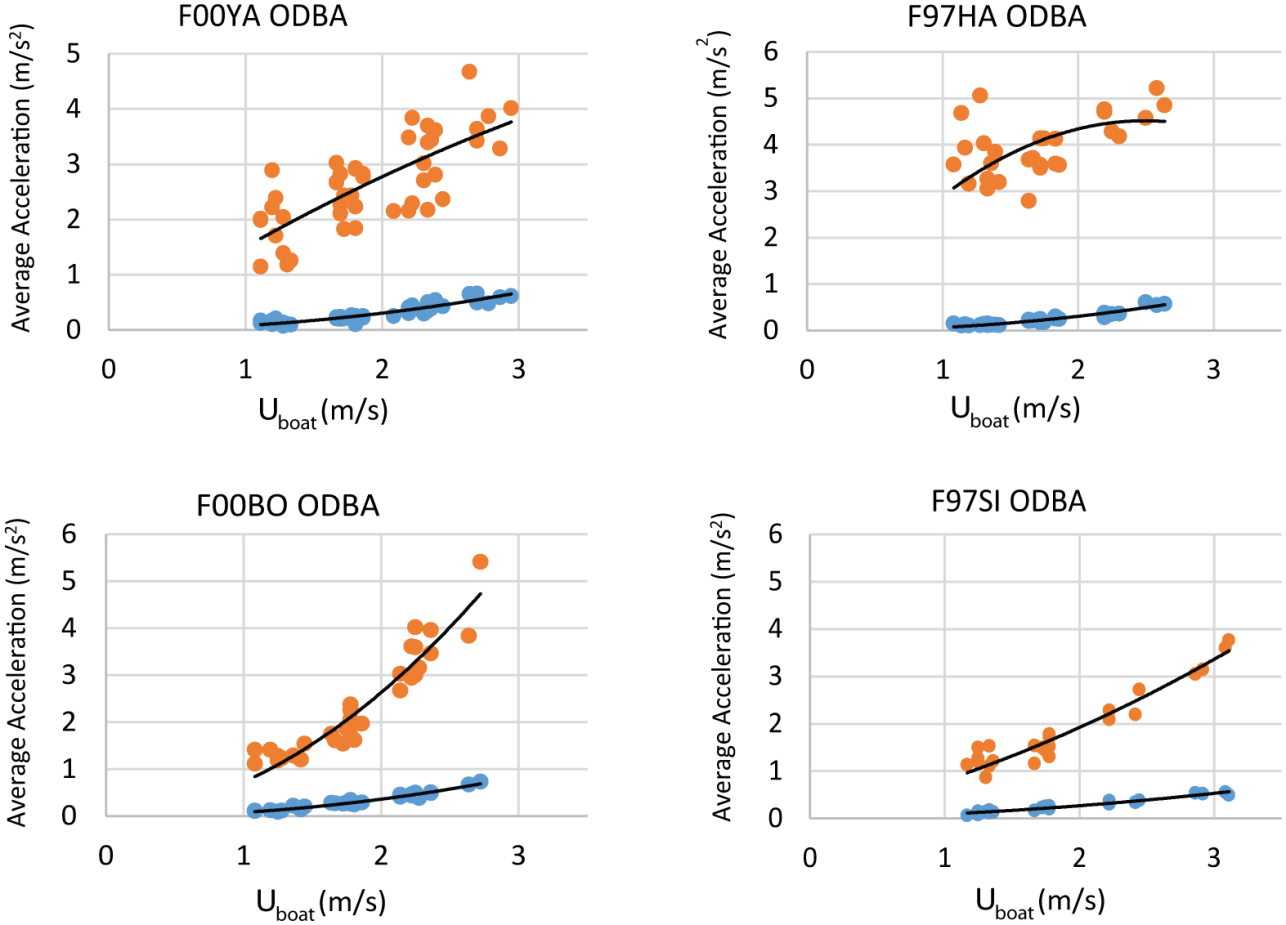
ODBA plots on a dive by basis for the four animals. APBA is shown for comparison in the lower curve on each plot.

The results for the ODBA metric averaged for all dives are given in Fig 11 plotted against speed. As can be seen, for two of the animals there is good agreement between swimming speed and the metric (F00BO, r^2^ = 0.90; F97SI, r^2^ = 0.93), but for the other two the correlation is relatively weak (F00YA, r^2^ = 0.57; F97HA, r^2^ = 0.04).

**Fig 12:**
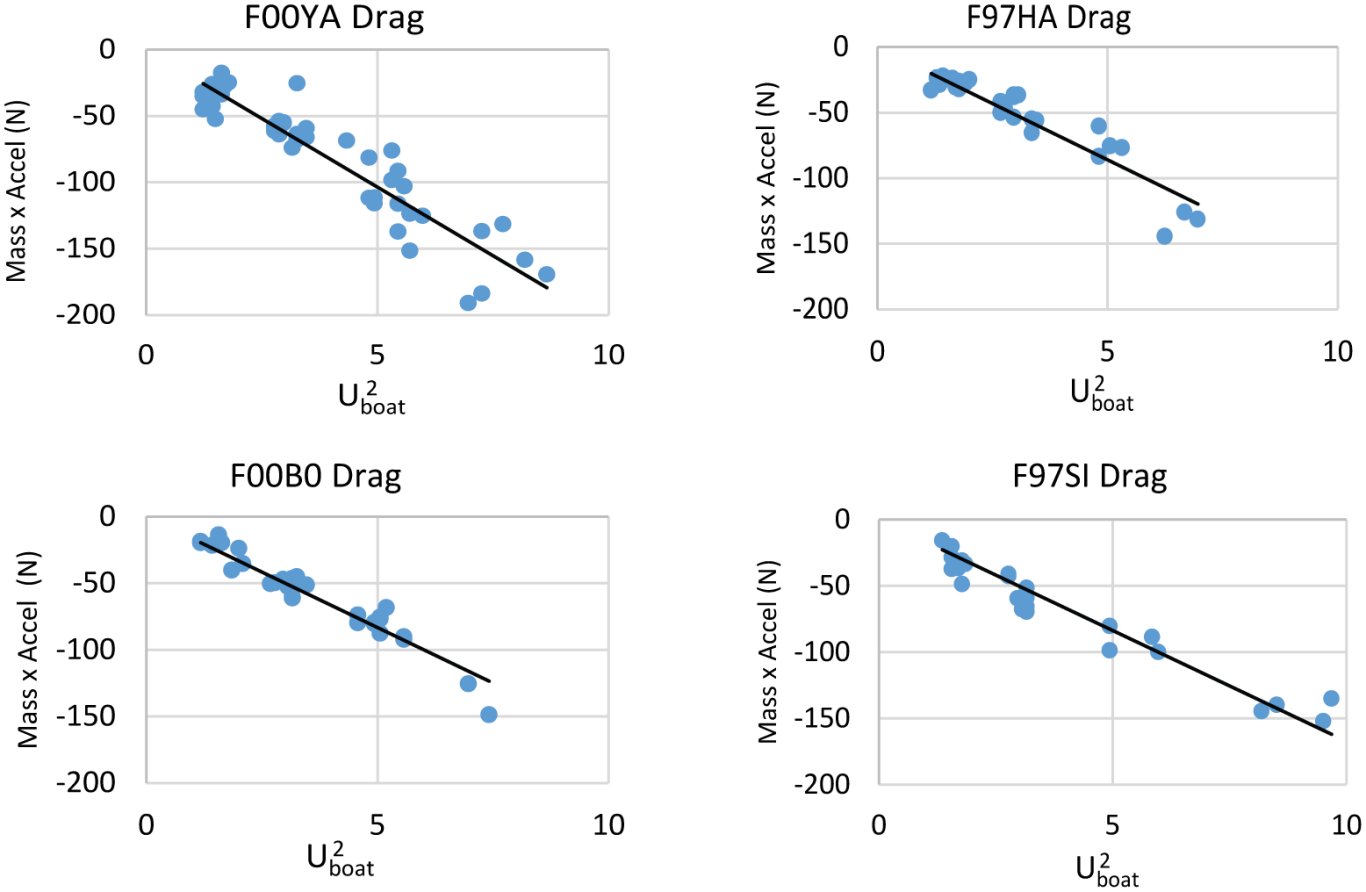
Time-averaged total drag, as calculated via Eg. 5, versus squared-boat speed.

**Fig 13:**
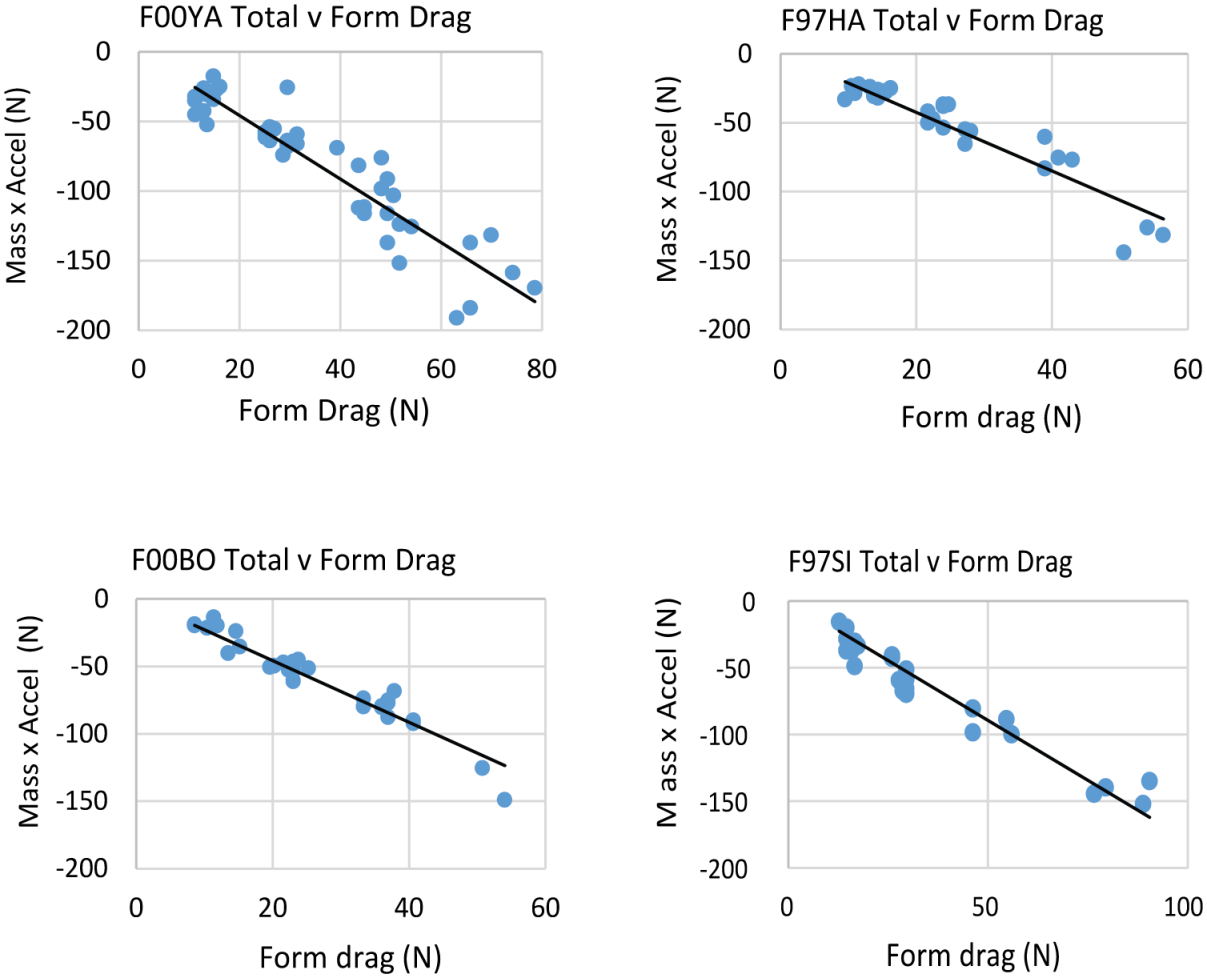
Total drag during the glide phase (Eq 5), versus form drag. The latter is represented by the *U*_*boat*_^*2*^ term in Eq 7.

Eq 5 gives the means for obtaining the total drag produced during the gliding phase. Fig 12 shows this drag to be mainly proportional to the square of the boat’s speed (*U*_*boat*_^*2*^). This does not necessarily imply the non-importance of harness drag since the value of the harness drag coefficient *k* (Eq 7) turns out to also depend on swimming speed. Fig 13 compares the total drag of Eq 5 with the form drag term, i.e., the *U*_*boat*_^*2*^ –term in Eq 7, as evaluated with γ = 1as for a swimmer moving at depth [27,28,31]. The results suggest the former is twice as large as the latter, i.e., as expected if the animals had been swimming at depth but with a draggy harness. This extra harness drag can be inferred by taking the difference between the ordinate and abscissa values in Fig 13, per Eqs 2 and 7.

**Fig 14:**
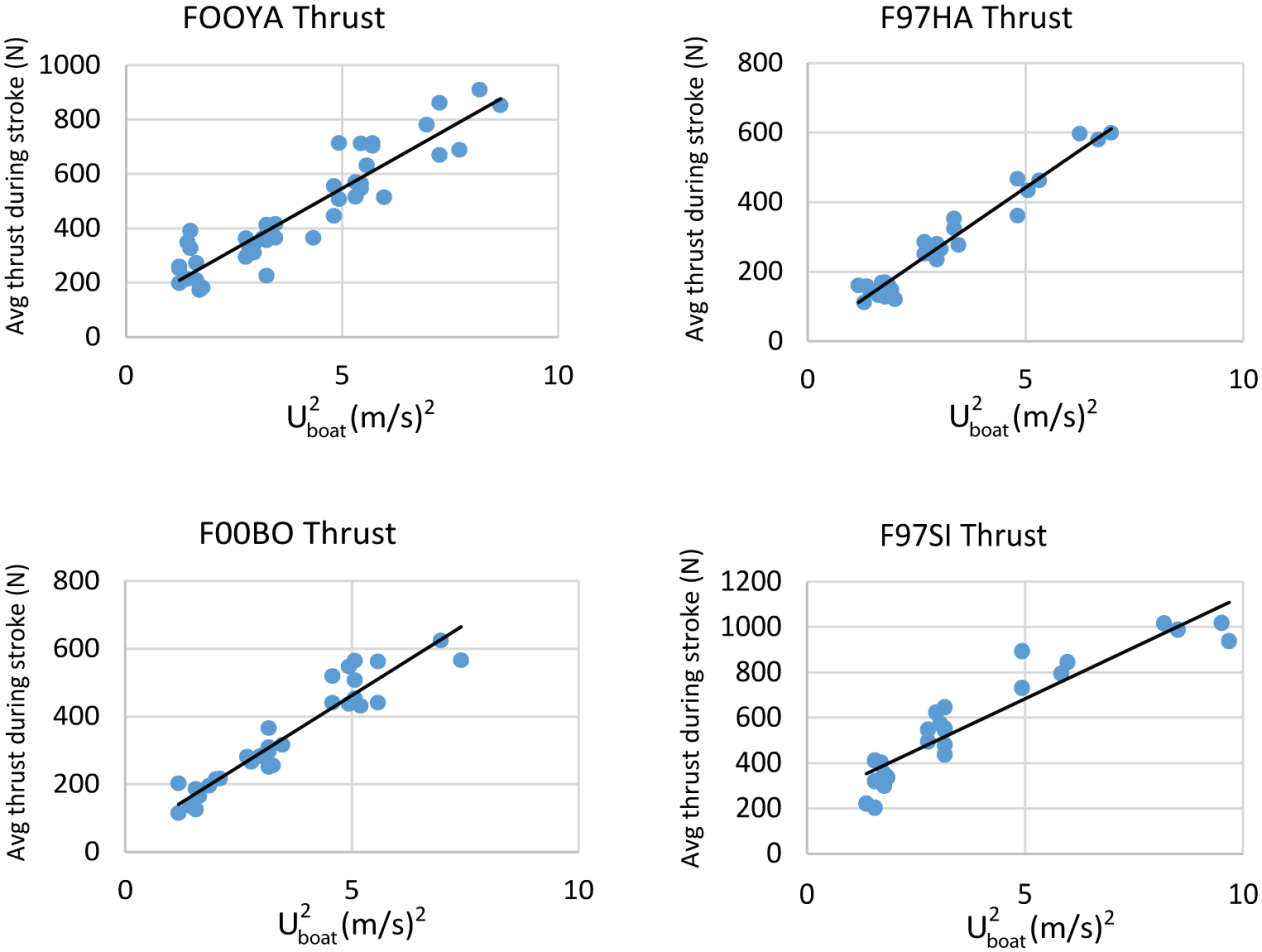
Average thrust during a stroke, as calculated via Eq 6, versus squared-boat speed.

**Fig 15:**
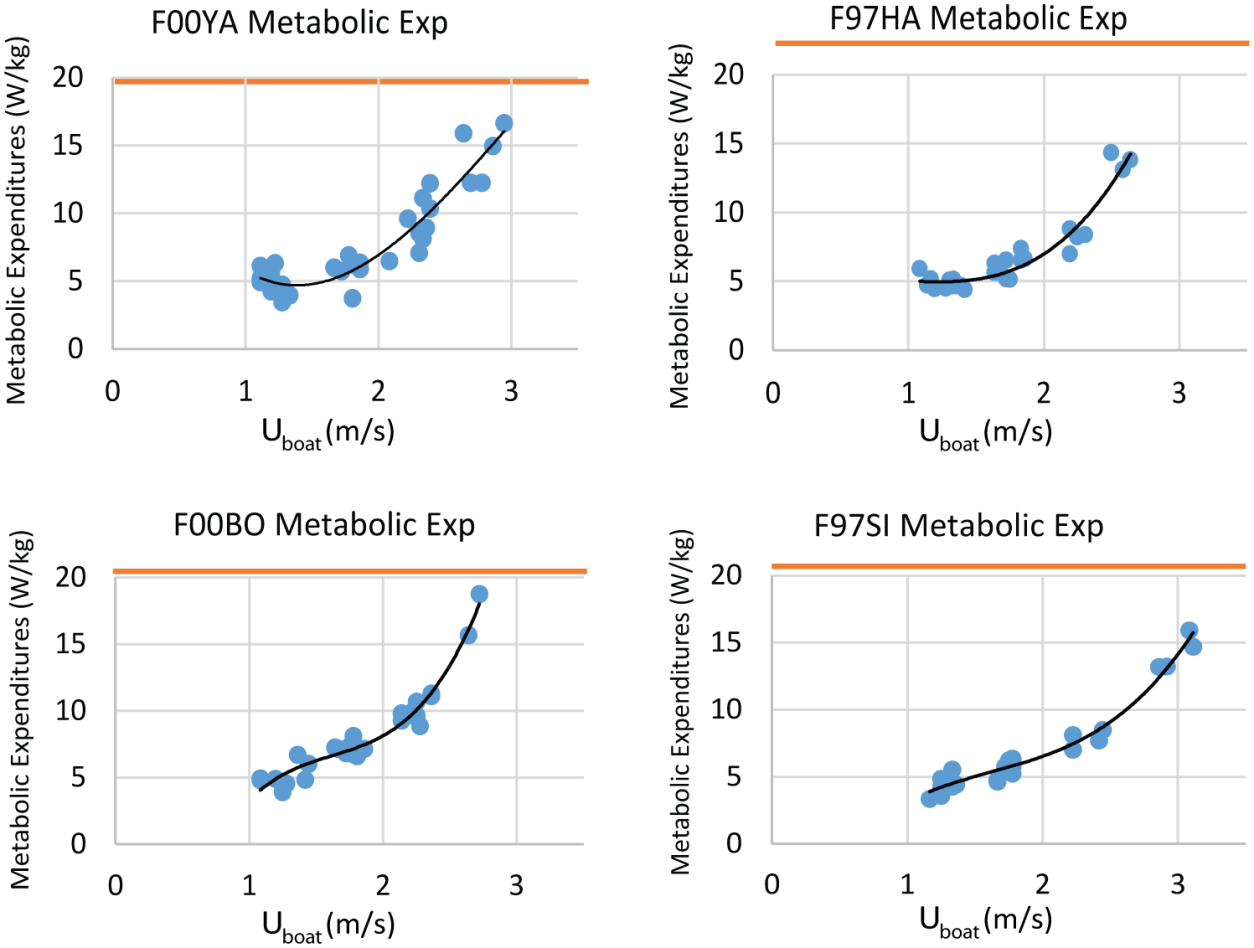
Total Metabolic Expenditures during one complete stroke-glide cycle (mass-specific) as a function of swimming speed. The estimated maximum metabolic rate is for terrestrial mammals [34] and is indicated by the orange lines.

Average stroke thrust calculated from Eq 6 is shown in Fig 14, highlighting an overall dependence on *U*_*boat*_^*2*^. From this, and from Eqs 8 and 9, the metabolic costs incurred during one full stroke-glide cycle are calculated and graphed in Fig 15. Estimated metabolic expenditures were noticeably less at the highest swimming speed, but close to maximal aerobic metabolic rates measured on terrestrial mammals [34] (F00BO, 90%, F00YA 85%, F97HA 67%, F97SI 82%).

## Discussion

Our results show that using gyroscope data combined with accelerometer data makes it possible to clearly identify individual flipper strokes for otariidae swimming in a straight line, and to estimate the gain in speed per stroke. It also makes it possible to calculate stroke rates. Stroke rate can be also calculated only with acceleration data using running mean or low-pass filters, but Fig 4 suggests that the results will be less reliable (compare Fig 5c with 5d).

We found a relatively weak correlation between stroke rates and swimming speed, which agrees with prior work by [18,32]. Feldkamp [18] noted that “frequency can vary by 50% at a single velocity”. Hindle et al. [32] found a negative correlation between ODBA/stroke and the stroke frequency at a given speed, suggesting that the animal can trade off stroke power for stroke frequency.

The average gain in speed per stroke was much more closely related to swimming speed than stroke frequency and the results show a speed gain per stroke increasing by a factor of 3–4 between the slowest and the fastest swimming speeds. Evidently, sea lions use an increase in stroke power to swim faster to a much greater extent than variation in stroke frequency. Fig 8 shows a large increase in the peak acceleration associated with strokes at higher speeds as well as a decrease in stroke duration. This suggests faster and possibly deeper strokes.

### APBA as a metric

Our results showed that accelerations during horizontal swimming at shallow depths correlated better with animal speed than did the ODBA method. This suggests that APBA may be a useful in estimating the energetic costs of locomotion at least during transits where depth changes are minimal.

In general, APBA will likely be most useful as a metric relating to swimming speed or energy expenditures for animals where propulsive accelerations can be clearly identified, such as with otariidae and penguins. Sea lions are almost ideal candidates for our method because their propulsive movements are characterized by brief strokes with glides between strokes making it easy to identify acceleration due to propulsion. Also, the metrics are likely to prove useful with animals where burst and glide swimming is characteristic. On the other hand, APBA would be useless for a body with continuous steady propulsion (such as a submarine) because no accelerations would occur at constant speeds, a problem that occurs also with ODBA and other related metrics.

### Near-surface and harness drag effects

Our results show the (total) drag generated in the runs to exceed body (form) drag (i.e., the *U*^*2*^ term in Eq 7) by about a factor of 2x. Part of the extra resistance may have to do with the wave drag effects that arise when swimming near the surface, and another part with the flutter of the aft harness strap and mounted tag. We also recognize that the harness may have impeded movement in ways other than drag.

The pressure sensor data showed the animals swimming at depths at which drag is minimal and where γ = 1 (Fig 9). Occasional small incursions to shallower depths of only 1.0 m were also recorded and would be enough to increase the value of the γ-parameter (Eq 7) from 1.0 to 1.2 [27,31,32]. Should this be the case, harness drag would amount to about 50% of the drag increase, in contrast to 100% of the increase if γ = 1.0 (swimming at depth).

Harness drag is the other plausible source here given the flutter recorded on the tag attached on the aft (caudal) strap (Fig 4). Hindle et al. [32] ran a number of trials on animals without and with a harness similar to those used in this study. In addition, the two animals were a subset of the three animals used here. They found a small decrease in the swimming strokes/ km (10% and 6%) per unit distance, when the animals swam without a harness. However, their aft strap did not carry hardware and thus could have been flutter-free. On the other hand, the mounting of the recording device shown in Fig 3 might have, via either vortex-shedding off-the tag’s back or tag upward-downward “kiting” motions, generated enough strap torsion and flagging to affect overall drag. Significant drag increase is plausible here since, in effect, strap flutter ends up increasing the amount of fluid turbulence found in an animal’s wake, and over a cross section area that now spans nearly all of a sea lion’s horizontally-projected maximum diameter.

### Potential for APBA to be used in dead reckoning

The APBA method has the potential to be useful in dead reckoning methods used to compute the trajectories of marine animals. A major problem with dead reckoning with tag data is that most tags do not record speed, although some do include a paddle wheel [35] or a propeller [13] for this purpose and flow noise has also been used to estimate speed with acoustic recording tags [7,36]. GIDA cannot be used to calculate speed by integrating accelerations over time; this approach would undoubtedly fail after a few seconds because of the inaccuracy of the low cost MEMS sensors. With more accurate sensors, integration would be possible over longer durations, but would still not be sufficient for dead-reckoning. APBA might be used to estimate speed using correlations, such as the ones we calculated, but we think that a more promising approach would be to use a propulsion-drag model, such as that proposed by [7] for humpback whales. As we have shown, gain in speed from brief strokes can be measured even with low accuracy sensors and this can be used to speed up an animal model, while estimated hydrodynamic drag can be used to slow the model if a drag coefficient can be approximated. This method should be especially effective for otariidae where it is possible to identify propulsive accelerations. However, we only have data thus far for straight-line swimming. Additional work will be required to use accelerations to estimate speed during the tight turns that are characteristic of sea lions when they are actively feeding. In addition, buoyancy forces must be taken into account especially when animals are diving vertically.

### Metabolic rate calculations

Considerable work has been done to estimate the energetic costs of diving while foraging for otadiids [2,33,37] and the energetic costs of steep dives can be calculated from speed estimates obtained using time and depth recorders. Our results provide a complementary method that should be useful in estimating energetic expenditures during horizontal swimming at depth. As shown in Fig 15, APBA-based calculations yield values that are consistent with these studies. Moreover, the values calculated in the high speed range (3m/s) are smaller but close to the maximal metabolic rate, i.e., ~ 85% maximum metabolic rate for three animals (based on terrestrial mammals [34]), and are consistent with the rapid fatigue exhibited by the sea lions during runs at those speeds.

### Assumptions and systematics

A number of assumptions are involved in using APBA to calculate metabolic expenditures. One is an assumption that the sensor is actually measuring acceleration at the center of mass of the animal; tags placed closer to the center of mass will result in better estimates of propulsive acceleration of the body mass. More peripheral placements may pick up transitory accelerations which are synchronized with stroking, but result in over or under-estimates of overall mass acceleration. In our case, the tag was held by the harness strap on the clavicles and there was considerable movement due to clavicle rotations and translations through the swimming stroke.

APBA ignores energy expended through angular accelerations and for this reason may underestimate propulsive effort especially when an animal is turning. Given a rigid body assumption, angular accelerations can be calculated in principle. However, sea lions have extremely flexible bodies and are able to conform their bodies to the radius of the turning circle when they turn, thereby minimizing additional drag and deceleration as body changes direction [38,39]. For other animals, especially turtles, the rigid body assumption is more accurate and in these cases estimating forces relating to angular accelerations may be feasible. More work will be required to determine the extent to which the energetic costs can be determined for frequently turning animals.

Another factor not accounted for by APBA is buoyancy. Overcoming either positive or negative buoyancy is a major cost of diving [40,41]. With horizontal swimming at shallow depths, as in our study, buoyancy will have less of an effect and it is possible that the animals in our study may have adjusted lung volume [42] so that they were roughly neutrally buoyant at the 1.5–2m swimming depths observed. In future work, it should be possible to combine APBA or similar metrics with buoyancy related costs to both estimate metabolic costs and create dead reckoned pseudo-tracks.

Inexpensive tags incorporating gyros promise to greatly improve the estimation of mechanical work done by animals when swimming compared with tags containing only accelerometer for otariidae. Even low cost tags with low cost gyros enable the magnitude of propulsive forces to be quantified, which should make it possible to use these measurements to more accurately estimate the metabolic costs of foraging.

## Appendix 1. Estimating dynamic acceleration using accelerometers and gyros

The tags were calibrated by holding the device steady in each of 6 orientations (upright and inverted) on each of the principle axes, and recording in each position for a few seconds. We then used the recorded data to obtain maxima and minima gravity values for each axis. Using these data, additive and multiplicative constants were calculated to scale the accelerometer signals between [-1,+1] on each axis.

These same fixed orientations were also used to estimate the fixed biases of the gyros because the tags were not being rotated. We nevertheless detected constant rotation rates of 5.03, 1.15 and 3.70 deg/s even though the gyros were not rotating in the fixed position. We subtracted these values from the gyro readings. Informal experiments using a lathe suggested that once these constants were subtracted angular velocity readings were accurate to within about 5%.

Dynamic linear tag acceleration *($\overset{⃑}{a}$)* can be calculated by subtracting the gravity vector from the measured acceleration vector if gravity can be estimated independently from accelerometer signals:

$$\vec{d}=\vec{a}-\vec{g}$$ (A1-1)

The problem is to obtain an estimate of the gravity vector g in tag coordinates. Our method is related to that of [21] and [24,43] and involves using the tag gyroscopes to maintain a continuously updated estimate of $\overset{⃑}{g}$. The method assume that the tag is held stationary for at least a second, when it is turned on in which case the accelerometers register a pure gravity vector signal ($\vec{a}\;=\;\vec{g}$). The average of ***$\overset{⃑}{a}$*** over one second can then be used to establish the starting point for the estimated gravity vector g⃑'. From this point forward, g⃑' is counter-rotated according to the signals from the gyroscopes at each time step. This results in g⃑' tracking $\overset{⃑}{g}$ continuously no matter how the tag is rotated.

The three-dimensional rotation of g’ is accomplished using quaternions. Quaternions are a method for representing rotations that avoids the problems with Euler angles (e.g. azimuth elevation and roll) [44]. Rotations are represented in quaternion form by a vector of four numbers, representing an axis of rotation and a rotation about that axis. A unit quaternion is a quaternion that has been normalized to have a length of 1.

Three axis rotations (*r*_*x*_, *r*_*y*_, *r*_*z*_) obtained from the gyros are converted to quaternion form as follows:

$$\theta =\sqrt{r_{x}^{2}+r_{y}^{2}+r_{z}^{2}}$$ (A1-2)

A unit quaternion is then constructed as:

q⇀=[cosθ2,u ⇀sinθ2] (A1-3)

where $\overset{⃑}{u}$ is a unit vector representing the axis of rotation constructed from the gyroscope measurements:

$$\vec{u}=\left[-\frac{r_{x}}{\theta },-\frac{r_{y}}{\theta },-\frac{r_{z}}{\theta }\right]$$ (A1-4)

Rotation of the virtual gravity vector is accomplished by pre-multiplying ${\overset{⃑}{g}}^{'}\;$ by the quaternion rotation and post-multiplying it by its inverse.

$${\vec{g}}_{t}^{\prime }={\vec{q}}_{t}{\vec{g}}_{t-1}^{\prime }{\vec{q}}_{t}^{-1}$$ (A1-5)

Given perfect gyroscope accuracy and no accumulation of numerical error, this method would be sufficient to maintain an accurate virtual gravity vector. The problem is that MEMS gyros are not perfect—they have low inherent accuracy and can have substantial constant rotation errors that drift over time [23,24].

A common way of compensating gyroscope errors is to use a complementary filter whereby high temporal resolution measurements can be corrected for drift using a measurement that does not drift (on average) over time. In this case the continuously updated estimation of g obtained with the gyros is corrected using the accelerometer signal, which averaged over time approximated g [24,43]. Animal accelerations are transitory and over the long term $\overset{⃑}{g}$ is approximated by $\overset{⃑}{a}$. To make use of this, measured acceleration $\overset{⃑}{a}$ can be combined in with the virtual gravity vector at each time step using a weighted average:

$$g_{t}{}^{\prime }=\left(1-\omega \right)g_{t}{}^{\prime }+\omega a_{t}$$ (A1-6)

where ω is a weight factor. The goal is to find a value of ω that eliminates drift. For example, we found *ω = 0*.*002* was effective at a 50 Hz sampling rate for our particular instrument. This method necessarily distorts the result somewhat—because steady prolonged accelerations will be underestimated as g⃑' drifts towards $\overset{⃑}{a}$, but the effect is minimal with transitory accelerations such as those caused by flipper strokes.

Dynamic acceleration is now calculated by:

$$\vec{d}=\vec{a}-\vec{g}\prime$$ (A1-7)

The MEMS gyros in the tag we employed displayed substantial constant errors of several degrees per s. To correct for this we measured the average bias with the tag held in a set of fixed positions and subtracted the result from all gyro measurements.

For an end to end test of the method we mounted the tag on a crank arm attached to the chuck of a large industrial lathe. This provided a test rig where constant dynamic accelerations could be generated and be easily calculated. For this simple case we could determine how accurately dynamic acceleration could be separated from static acceleration.

The magnitude of the centripetal acceleration ($\overset{⃑}{d}$) is given by

$$\left\|\vec{d}\right\|=w^{2}r$$

where w is the angular speed in radians/s and r is the radius.

Using the circuit board diagram provided by the manufacturer we determined the location of the accelerometer chip within the tag and mounted, the tag on a custom made crank arm so that the accelerometers were placed at 9 and 18 cm eccentricities to the lathe axis of rotation.

We set the lathe rotating with nominal speeds of 30 and 60 rpm in both clockwise and counter clockwise directions. To measure the actual speed of rotation we determined the period for 10 cycles using the accelerometer measurements. Table 1 shows the magnitude of the calculated GIDA values. The estimate of centripetal acceleration is the GIDA component in the direction of the axis rotation. The results are based on average values for 16 seconds of steady rotation.

The result are given in Table 3. They reveal overestimates of the larger dynamic accelerations and underestimates the smaller dynamic accelerations. The absolute size of the estimation errors increases with the acceleration.

**Table 3:** Results from the lathe test rig with estimated centripetal accelerations compared to actual centripetal accelerations. Standard deviations are given in brackets.

Rotationspeed (rad/s)	Radius (m)	Centripetalaccelerationm/s^2^	GIDA m/s^2^	Estimated centripetal acceleration (m/s^2)^	Centripetalerror m/s^2^
0.0	-	0.0	0.159 (0.03)	0.133 (0.02)	0.133
-2.605	0.09	0.611	0.539(0.18)	0.467 (0.21)	-0.144
	0.18	0.618	0.643(0.27)	0.488 (0.33)	-0.155
2.618	0.09	1.222	1.122 (0.19)	1.051 (0.39)	-0.169
	0.18	1.234	1.184 (0.40)	1.073 (0.19)	-0.161
-6.163	0.09	3.440	3.740 (0.54)	3.693 (0.55)	0.253
	0.18	3.343	3.740 (0.54)	3.647 (0.66)	0.309
6.094	0.09	6.881	7.382 (1.61)	7.334 (0.59)	0.453
	0.18	6.861	7.358 (0.82)	7.257 (0.81)	0.396

In addition, as mentioned above, MEMS gyros can drift over time and an incorrect gyro reading can result in a false estimate of dynamic accelerations. In our algorithm development we observed stretches of the track where the mean acceleration was not centered on zero (as it should be for a steadily swimming animal). To correct for gyro bias drift we subtracted the estimated dynamic acceleration from a running mean of the estimated dynamic acceleration as a final processing step. We found a 30 sec running average to be effective. The long duration is designed to not interfere with the detection short term dynamic events, such as propulsive flipper strokes. It is also important to note that this additional stage of processing should not be needed since the complementary filter is intended to correct for gyro drift and other inaccuracies. An alternative would be to simply increase the weighting of the accelerometer signal. Never the less, we believe that the method has value because it allowed us to use a smaller weight and thereby resolve transient events more accurately and that the problem (of non-zero mean acceleration) only occurred occasionally. In addition, using a 30 second running average will have no significant effect on our basic results. We suggest that this final stage of processing should be considered to be optional and not part of the basic method.

Source code together with both original and processed data files are provided as S1–S5 Data.

## Appendix 2. Physics of body acceleration, work and drag

Speed modeling

The forces and metabolic expenditures discussed in this paper are based on time-averaged accelerations and forward speeds collected from the tag data. Several assumptions are made here, namely with regards to the periodicity of the GIDA signal and integrated variables over *all* stroke-glide cycles, as well as the averaged otariid speed (and squared speed). As intuitive as they may appear, these need to be justified.

As shown in Fig 7c and 7d, the tag signal is quasi-periodic, i.e., achieving strict periodicity only over a few cycles, most probably as a result of the animals’ change of behavior during a run. In a truly periodic swim, a sea lion would have achieved the same maximum forward speed (*U*_*max*_) at the end of each stroke, and the same minimum speed (*U*_*min*_) at the end of each glide (Fig 7d). Similarly, durations *t*_*s*_, *t*_*g*_ and *t*_*f*_ would have been the same over all cycles. But the variations from strict periodicity are small enough, and as a result permit the assessment via simple modeling of the errors incurred when replacing time-averaged speeds by boat speeds (and powers of thereof). The models used here are the linear function represented by the red dashed lines in Fig 7d and expressed mathematically as follows:

$$U(t)=\frac{\left(U_{\text{max}}-U_{\text{min}}\right)}{t_{s}}t+U_{\text{min}}\;\left(\text{stroking phase}\right)$$ (A2-1)

$$U(t)=\frac{-\left(U_{\text{max}}-U_{\text{min}}\right)}{t_{g}}\left(t-t_{s}\right)+U_{\text{max}}\;\left(\text{gliding phase}\right)$$ (A2-2)

Overall, these functions provide a good -to- acceptable approximations over most of the recorded stroke-glide cycles. More importantly, they allow the exact calculation of their time averages via integration per the procedure defined in Eq A2-7 below, to result in

$$U_{boat}\equiv \langle U(t)\rangle =\frac{1}{2}\left(U_{\text{max}}+U_{\text{min}t}\right)$$ (A2-3)

$$\langle U{\left(t\right)}^{2}\rangle =U_{boat}{}^{2}+\frac{{\left(U_{\text{max}}-U_{\text{min}}\right)}^{2}}{12}\approx U_{boat}{}^{2}$$ (A2-4)

Interestingly, these results are the same in both the stroke and glide phases. In Eq A2-3, the boat’s speed is *defined* as identical to the sea lion’s averaged speed, a result which is then used in Eq A2-4. The last step in the right-hand-side of Eq A2-4 follows from the second term being much smaller than *U*_*boat*_^*2*^, namely, 0.19 m^2^/s^2^ << 5.56 m^2^/s^2^, as can be verified in Fig 7d and 7e. (Note: the factor 1/12 in Eq A2-4 is a coefficient derived exactly from the time average of *U*^*2*^*(t)*, which during the stroke phase can be written as follows: ${\langle U{\left(t\right)}^{2}\rangle }_{ts}=\frac{1}{t_{s}}\int_{0}^{ts}{\left[\left(\frac{2\Delta U}{t_{s}}\right)t+\left(U_{boat}-\Delta U\right)\right]}^{2}dt$ with *ΔU = (U*_*max*_*−U*_*min*_*)/2* and *U*_*min*_ = *U*_*boat*_*−ΔU*. The factor readily appears after carrying out the square and integration).

Force and acceleration time-averaging

The major energy expenditures in horizontal straight-line swimming come from overcoming fluid drag. In a flipper stroke where the propulsion force *T* is generated, an animal increases its speed from *V*_*min*_ to *V*_*max*_, followed by the glide where the speed declines from *V*_*max*_ to *V*_*min*_ due to fluid drag (*D*). Herein the equations of the forward motions corresponding to the foreflipper stroke and glide phases are:

$$\left(M_{animal}+M_{entrained}\right)a(t)=T(t)-D(t)\;\left(\text{stroking phase}\right)$$ (A2-5)

$$\left(M_{animal}+M_{entrained}\right)a(t)=-D(t)\left(\text{gliding phase}\right)$$ (A2-6)

The functions *a*, *D and T* are shown as explicitly time-dependent, and *a(t)* can be either negative or positive while *T(t)* and *D(t)* are positive only. Time-averaging both sides of these equations is carried out via the following definition, here applied to an arbitrary function *F(t)* over time interval *T*:

$${\langle F\rangle }_{T}\equiv \frac{1}{T}\int_{0}^{T}F(t)dt$$ (A2-7)

Carried out over the relevant time intervals defined in Fig 7c, this procedure yields Eqs 2–4. Since the intervals are such that *t*_*f*_ = *t*_*s*_ *+ t*_*g*_, time-averaging the acceleration results in

$${\langle a\rangle }_{tf}=\frac{1}{t_{f}}\int_{0}^{tf}a(t)dt=\left[\frac{t_{s}}{t_{f}}\frac{1}{t_{s}}\int_{0}^{ts}a(t)dt+\frac{t_{g}}{t_{f}}\frac{1}{t_{g}}\int_{ts}^{tf}a(t)dt\right]=\frac{t_{s}}{t_{f}}{\langle a\rangle }_{ts}+\frac{t_{g}}{t_{f}}{\langle a\rangle }_{tg}=0$$ (A2-8)

Here <a>_tf_ is set to zero following the fact that the sea lion is following a boat cruising at fixed speed. Again, this constraint is only an approximation at the level of individual stroke-glide cycles but holds over many such cycle. Referring to the sample velocity profile shown in Fig 7d, and by definition of the Averaged Propulsive Body Acceleration, one has *(APBA) t*_*f*_ = *(V*_*max*_*−V*_*0*_*)*. This same speed gain is also equal to *(V*_*max*_*−V*_*0*_*) = <a>*_*ts*_ *t*_*s*_ = - *<a>*_*tg*_ *t*_*g*_, which along Eq A2-8 yields Eq 1.

Another important relationship is that of the average propulsive force, i.e., averaged over the stroke duration (*t*_*s*_) and also over the duration of a full stroke-glide cycle (i.e., *t*_*s*_ *+ t*_*g*_). Noting that *<T>*_*tg*_ = *0* one has

$$t_{f}{\langle T\rangle }_{tf}=t_{s}{\langle T\rangle }_{ts}$$ (A2-9)

Estimation of the time-averaged drag proceeds along similar lines and results in

$${\langle D\rangle }_{tf}=\frac{t_{s}}{t_{f}}{\langle D\rangle }_{ts}+\frac{t_{g}}{t_{f}}{\langle D\rangle }_{tg}$$ (A2-10)

We note that Eqs A2-9 and A2-10 are general rather than specific to Eqs A2-1 and A2-2. The next step involves more specific modeling of the drag:

$$D(t)=D_{body}(t)+D_{straps}(t)$$ (A2-11)

$$D_{body}(t)=\gamma \frac{1}{2}\rho S_{wetted}C_{D}{}^{body}U^{2}(t)$$ (A2-12)

$$D_{straps}(t)=k\rho (TAD)U(t)\omega (t)$$ (A2-13)

The input coefficients are the same as those of Eq 7. Time-averaging the drag terms with the speed model of Fig 7d Eqs A2-1–A2-4 implies that the average drag is the same in each stroke and glide phase, i.e., and *<D>*_*tg*_ = *<D>*_*ts*_. Note that the flutter coefficient was assumed as constant during a stroke-glide cycle and over the boat speed range considered.

Merging these results also yield Eqs 5 and 6 as follows. Using *(APBA) t*_*f*_ = *<a>*_*ts*_ *t*_*s*_ = - *<a>*_*tg*_ *t*_*g*_ and *<D>*_*tg*_ = *<D>*_*ts*_ in Eq 1 leads to

$$\begin{array}{l} {\langle D\rangle }_{tg}=(-1)\left(M_{animal}+M_{entrained}\right)(-1)\left(APBA\right)\frac{t_{f}}{t_{g}} \end{array}$$ (A2-14)

This is a result that simplifies into Eq 5. On the other hand, using them in Eq 2 gives

$${\langle T\rangle }_{ts}=\left(M_{animal}+M_{entrained}\right)\frac{t_{f}}{t_{s}}\left(APBA\right)+{\langle D\rangle }_{tg}$$ (A2-15)

Using Eqs A2-9 and A2-10 in Eq A2-15 then results in

$${\langle T\rangle }_{tf}\frac{t_{f}}{t_{s}}=\left(M_{animal}+M_{entrained}\right)\cdot \left(APBA\right)\cdot \left[\frac{t_{f}}{t_{s}}+\frac{t_{f}}{t_{g}}\right]$$ (A2-16)

which simplifies to the following (and Eq 6) after using the definition *t*_*f*_ = *t*_*s*_ *+ t*_*g*_ (Fig 7)

$$\begin{array}{l} {\langle T\rangle }_{tf}=\left(M_{animal}+M_{entrained}\right)\cdot \left(APBA\right)\cdot \left[\frac{t_{f}}{t_{g}}\right]\\ =\left(M_{animal}+M_{entrained}\right)\cdot \left(APBA\right)\cdot \left[\frac{t_{f}}{t_{f}-t_{s}}\right] \end{array}$$ (A2-17)

Spreadsheets applying these methods to the data are provided as supplementary materials (S1 Data).

## Supporting Information

S1 Data This zipped folder contains a set of summary spreadsheet files, two for each animal. The files with names ending in NewAnalysis contain the data used to construct the APBA and ODBA plots. The files ending with “JP edits” contain calculations relating to drag and energy expenditures. Also included is C++ source code required to apply the GIDA method to the raw data.
(ZIP)

S2 Data Unpacked raw openTag files are provided for animal F00BO,. Also, a set of csv files, one for each dive included in the study. Each file contains a 50Hz time series. Provided are calibrated accelerometer and gyro measurements, together with the estimated gravity vector, and the estimated dynamic accelerations by the different methods. The small files with the suffix.txt are configuration files used in C++ processing.
(ZIP)

S3 Data Unpacked raw openTag files are provided for animal F97HA. Same components as S2.
(ZIP)

S4 Data Unpacked raw openTag files are provided for animal F97SI. Same components as S2.
(ZIP)

S5 Data Unpacked raw openTag files are provided for animal F97SI. Same components as S2.
(ZIP)

## References

[1] Charnov EL, Optimal foraging, the marginal value theorem. Theor Popul Biol. 1976; 9: 129–136. 1273796 10.1016/0040-5809(76)90040-x

[2] Williams TM. The evolution of cost efficient swimming in marine mammals: limits to energetic optimization. Philos Trans R Soc Lond B Bio Sci. 1999; 354: 193–201.

[3] Wilson RP, White CR, Quintana F, Halsey LG, Liebsch N, Martin GR, et al Moving towards acceleration for estimates of activity-specific metabolic rate in free-living animals: the case of the cormorant. J Anim Ecol. 2006;75: 1081–1090. 16922843 10.1111/j.1365-2656.2006.01127.x

[4] Gleiss AC, Wilson RP, Shepard LC. Making overall dynamic body acceleration work: on the theory of acceleration as a proxy for energy expenditure. Methods Ecol Evol. 2011;2: 23–33.

[5] Johnson MP, Tyack PL. A digital acoustic recording tag for measuring the response of wild marine mammals to sound. IEEE J Oceanic Eng. 2003 1;28(1):3–12.

[6] Shepard ELC, Wilson RP, Halsey LG, Quintana F, Gómez Laich A, Gleiss AC, et al Derivation of body motion via appropriate smoothing of acceleration data. Aquat Biol. 2008;4: 235–241.

[7] Ware C, Friedlaender AS, Nowacek DP. Shallow and Deep Lunge Feeding of Humpback Whales in Fjords of the West Antarctic Peninsula. Mar Mamm Sci. 2011;227: 587–605.

[8] Tanaka H, Takagi Y, Naito Y. Swimming speeds and buoyancy compensation of migrating adult chum salmon *Onchorhynchu keta* revealed by speed/depth/acceleration data logger. J Exp Biol. 2001;204:3895–3904. 11807107 10.1242/jeb.204.22.3895

[9] Martin López LM, Miller PJ, de Soto NA, Johnson M. Gait switches in deep-diving beaked whales: biomechanical strategies for long-duration dives. J Exp Biol. 2015; 218(9):1325–1338. 25954042 10.1242/jeb.106013

[10] Halsey LG, Shepard ELC, Quintana F, Gomez Laich A, Green JA, Wilson RP. The relationship between oxygen consumption and body acceleration in a range of species. Comp Biochem Physiol A: Mol Integr Physiol. 2009 2 28;152(2):197–202. 18854225 10.1016/j.cbpa.2008.09.021

[11] Qasem L, Cardew A, Wilson A, Griffiths I, Halsey LG, Shepard EL, et al Tri-axial dynamic acceleration as a proxy for animal energy expenditure; should we be summing values or calculating the vector? PloS One, 2012;7(2), e31187 doi: 10.1371/journal.pone.0031187 22363576 10.1371/journal.pone.0031187 PMC3281952

[12] Gleiss AC, Dale JJ, Holland KN, Wilson RP. Accelerating estimates of activity-specific metabolic rate in fishes: testing the applicability of acceleration data-loggers. J Exp Mar Biol Ecol. 2010;385: 85–91.

[13] Mitani Y, Sato K, Ito S, Cameron MF, Siniff DB, Naito Y. A method for reconstructing three-dimensional dive profiles of marine mammals using geomagnetic intensity data: results from two lactating Weddell seals. Polar Biol. 2003;26(5): 311–317.

[14] Wilson RP, Wilson MP. Dead reckoning: a new technique for determining penguin movements at sea. Meeresforschung. 1988;32(2):155–8.

[15] Noda T, Okuyama J, Koizumi T. Arai N, Kobayashi M. Monitoring attitude and dynamic acceleration of free-moving aquatic animals using a gyroscope. Aquatic Biol. 2012;16: 265–276.

[16] Fish FE, Innes S, Ronald K. Kinematics and estimated thrust production of swimming harp and ringed seals. J Exp Biol. 1988;137: 157–173. 3209965 10.1242/jeb.137.1.157

[17] English AW. Limb movements and locomotor function in the California sea lion (Zalophus californianus). J Zool. 1976;178(3): 341–364.

[18] Feldkamp SD. Forflipper propulsion in the California sea lion, Zalophus Californianus. J Zool. 1987; 212(1): 43–57.

[19] Feldkamp SD. Swimming in the California sea lion: morphometrics, drag and energetics. J Exp Biol. 1987;131(1), 117–135. 3694112 10.1242/jeb.131.1.117

[20] Stelle LL, Blake RW, Trites AW. Hydrodynamic drag in Steller sea lions (Eumetopias jubatus). J Exp Biol. 2000; 203; 1915–1923. 10821748 10.1242/jeb.203.12.1915

[21] Luinge HJ, Veltink PH. Measuring orientation of human body segments using miniature gyroscopes and accelerometers. Med Biol Eng Comput. 2005 4 1;43(2):273–282. 15865139 10.1007/BF02345966

[22] Noda T, Kawabata Y, Arai N, Mitamura H, Watanabe S. Animal-mounted gyroscope/accelerometer/magnetometer: In situ measurement of the movement performance of fast-start behaviour in fish. J Exp Mar Biol Ecol. 2014 2 28;451:55–68.

[23] Euston M, Coote P, Mahoney R, Kim J, Hamel TA complementary filter for attitude estimation of a fixed-wing UAV. In Proc. IEEE/RSJ International Conference on Intelligent Robots and Systems. Nice, 2008;340–345.

[24] Fourati H, Noureddine N, Afilal L, Handrich Y. Posture and body acceleration tracking by inertial and magnetic sensing: Application in behavioral analysis of free-ranging animals. Biomed Signal Process Control. 2011;6(1): 94–104.

[25] Webb PW, Cotel AJ, Turbulence: Does Vorticity Affect the Structure and Shape of Body and Fin Propulsors? Integr Comp Biol. 2010; 50(6): 1155–1166 doi: 10.1093/icb/icq020 21558264 10.1093/icb/icq020

[26] Dole CE. Flight Theory and Aerodynamics-A Practical guide for operational safety. 1981 Wiley.

[27] Fish FE. Power output and propulsive efficiency of swimming bottlenose dolphins (Tursiops truncatus). J Exp Biol. 1993;185(1), 179–193.

[28] Fish FE. Comparative kinematics and hydrodynamics of odontocete cetaceans: Morphological and ecological correlates with swimming performance. J Exp Biol. 1998;201(20):2867–2877. 9866875

[29] Williams TM. Approaches for the study of exercise physiology and hydrodynamics in marine mammals. Approaches to marine mammal energetics. 1987(1):127–45.

[30] Kooyman GL. 1989 Diverse Divers:Physiology and Behavior Zoophysiology Series, Ed: Johansen K, Farner DS, Vol. 23 New York: Springer-Verlag.

[31] Hertel H. Structure, Form, Movement. New York: Reinhold; 1966.

[32] Hindle AG, Rosen DA, Trites AW. Swimming depth and ocean currents affect transit costs in Steller sea lions Eumetopias jubatus. Aquat Biol. 2010 8;10(2):139–48.

[33] Costa DP, Gales NJ. Energetics of a benthic diver: seasonal foraging ecology of the Australian sea lion, Neophoca cinerea. Ecol Monog. 2003;73(1): 27–43.

[34] Weibel ER, Hoppeler H. Exercise-induced maximal metabolic rate scales with muscle aerobic capacity. J Exp Biol. 2005;208: 1635–1644. 15855395 10.1242/jeb.01548

[35] Wilson RP, Shepard ELC, Liebsch N. Prying into the intimate details of animal lives: use of a daily diary on animals. Endangered Species Res. 2008;4: 123–137.

[36] Goldbogen JA, Calambokidis J. Shadwick RE, Oleson EM, McDonald MA, Hildebrand JA. Kinematics of foraging dives and lunge-feeding in fin whales. J Exp Biol. 2006;209: 1231–1244. 16547295 10.1242/jeb.02135

[37] Fahlman A, Wilson R, Svärd C., Rosen DA, Trites AW. Activity and diving metabolism correlate in Steller sea lion Eumetopias jubatus. Aquat Biol. 2008;2, 75–84.

[38] Fish FE, Hurley J, Costa DP. Maneuverability by the sea lion Zalophus californianus: turning performance in an unstable body design. J Exp Biol. 2003;206: 6677–674. 10.1242/jeb.00144 12517984

[39] Cheneval O, Blake RW, Trites AW, Chan KH, Turning manoeuvres in Steller sea lions (Eumetopias jubatus). Mar Mamm Sci. 2007;23: 94–109.

[40] Miller PJ, Johnson MP, Tyack PL, Terray EA. Swimming gaits, passive drag and buoyancy of diving sperm whales Physeter macrocephalus. J. Exp Biol. 2004;207(11): 1953–1967. 15107448 10.1242/jeb.00993

[41] Sato K, Mitani Y, Cameron MF, Siniff DB, Naito Y. Factors affecting stroking patterns and body angle in diving Weddell seals under natural conditions. J Exp Biol. 2003;206(9), 1461–1470. 12654885 10.1242/jeb.00265

[42] Fahlman A, Hastie GD, Rosen DAS, Naito Y, Trites AW, Bouyancy does not affect diving metabolism during shallow dives in Steller sea lions *Eumetopias jubatus*. Aquat Biol. 2008; 3: 147–154.

[43] Fourati H, Manamanni N, Afilal L, Handrich Y. Rigid body motions capturing by means of wearable inertial and magnetic MEMS sensors assembly: toward the reconstitution of the posture of free ranging animal in Bio-logging In: Novel Microelectronics: Technologies and Systems Applications, CRC Press; 2013 pp. 324–466.

[44] Shoemake K. Animating rotation with quaternion curves. ACM SIGGRAPH Comput Graph Proc Annu Conf Ser. 1985;19: 245–254.

